# Adsorption-Based Methane Removal from Dilute Non-fossil Sources: Process Concept and Sorbent Screening

**DOI:** 10.1021/acs.iecr.5c05045

**Published:** 2026-08-05

**Authors:** Kian Karimi, Matteo Gazzani

**Affiliations:** † Copernicus Institute of Sustainable Development, Faculty of Geosciences, Utrecht University, Princetonlaan 8a, 3584 CB Utrecht, The Netherlands; ‡ Inorganic Membranes and Membrane Reactors, Sustainable Process Engineering, Chemical Engineering and Chemistry, Eindhoven University of Technology, Eindhoven 5600, The Netherlands

## Abstract

Methane removal from dilute emission sources is gaining attention as a climate mitigation strategy targeting hard-to-abate non-fossil emissions; however, its process-level feasibility remains largely unexplored. This work develops an equilibrium-based, zero-dimensional (0D) vacuum-temperature swing adsorption (VTSA) model that enables rapid, physics-informed simulation of CH_4_–CO_2_–N_2_ separations and supports high-throughput sorbent screening (using here the NIST/ARPA-E adsorption data). The model is verified against a detailed one-dimensional (1D) rate-based model and coupled with a process concept incorporating subambient adsorption and heat integration. Three representative concentration regimes were investigated: semiclosed dairy barns (100 ppm of CH_4_), open barns and biogenic distributed sources (25 ppm of CH_4_), and direct-air-capture-like conditions (2 ppm of CH_4_). Across these cases, different existing materials were identified, including activated carbons, MOFs, and zeolites. Methane purities range from few percentage points to double-digit values, where the latter need to be confirmed with improved experimental characterization in the low partial pressure regime. Exergy consumptions are significant in all cases, with values starting from around 60 MJ/kgCH_4_, which, however, corresponds to around 2 MJ/kgCO_2_eq on a 100 years global warming potential basis. Direct methane removal from air appears to be extremely challenging, requiring a breakthrough on both material and process. These results indicate that methane capture from agriculture dilute sources is potentially viable with existing sorbents using a VTSA cycle specifically engineered for the purpose. Yet, they also highlight that further experimental characterization, validation, and detailed process modeling, for example, with rate-based 1D models and competitive isotherms, are needed to confirm these results and advance this separation process.

## Graphical abstract

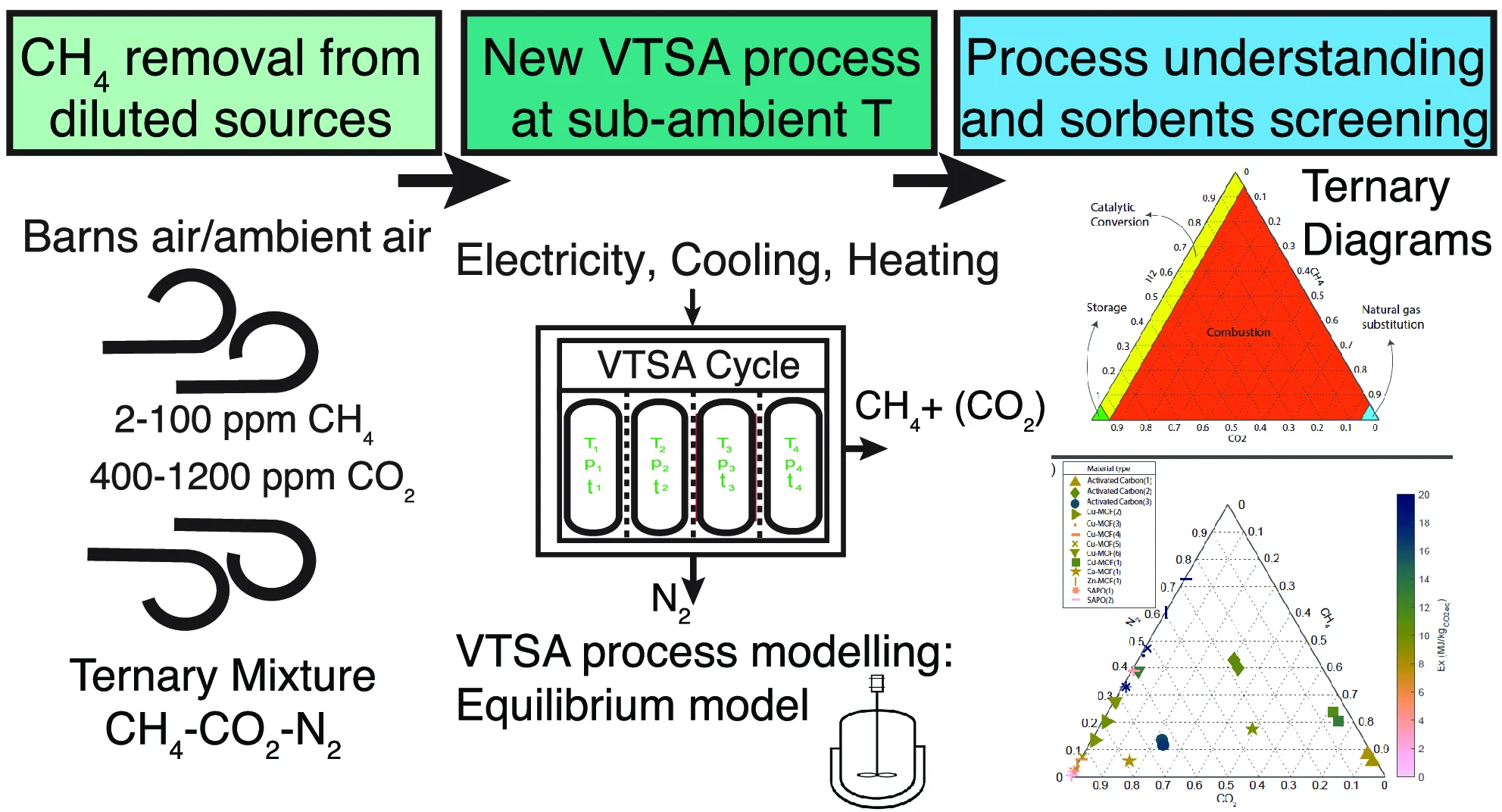

## Introduction

On 23rd October 2015, the collapse of a wellhead at the natural gas storage basin in Aliso Canyon, northeast of Los Angeles, led to the release of approximately 97 100 t of methane to the atmosphere. About 1700 homes and two schools were evacuated. The gas leak had the largest impact on climate from a single incident on US records, compromising California’s effort to reduce greenhouse gas (GHG) emissions in 2015.^1^ The incident is yet more evidence that we lack solutions for tackling CH_4_ emissions.

Methane is a potent short-lived greenhouse gas that has contributed to 0.5 °C of warming until the 2010s, which corresponds to two-thirds of the CO_2_ contribution.^2,3^ Despite the short atmospheric lifetime (approximately 12 years) due to oxidation by a hydroxyl radical, methane’s molecular structure contributes to a high radiative efficiency, resulting in a global warming potential of around 28 over a period of a hundred years, >80 on a 20 year basis.^3^ Due to human-induced emissions (i.e., including feedback effects), the global mean atmospheric concentration of methane has currently reached approximately 2 ppm, more than 2.5 times preindustrial levels. It might therefore not be surprising that, in order to stay below 2 °C global warming and to mitigate near-term warming, emissions from short-lived climate pollutants, and particularly methane, need to be cut.^4–6^ This is particularly relevant to prevent climate extremes and facilitate mitigation and adaptation. Accordingly, more than 150 nations have endorsed the global methane pledge.^7^ However, as shown in Figure 1, methane concentration in the atmosphere has been relentlessly growing during the last ten years, in line with IPCC scenarios for high global temperature increase.^8,9^ Unlike CO_2_, which is mainly emitted by burning fossil fuels, methane emissions sources are diverse and dispersed: According to the most recent work of the Global Methane Budget, the annual global methane emissions are estimated around [575–669] Mt/year [bottom-up to top-down budget], while total sinks account for [633–554] Mt/year. Of the total emissions, around 40% originates from natural sources, with the remaining 60% due to human-related activities: agricultural and waste [211–228], fossil fuel production and use [120–115], and biomass and biofuel burning [28–27]. Overall, this results in a net increase of methane in the atmosphere of around 36–21 Mt/year.

**Figure 1:**
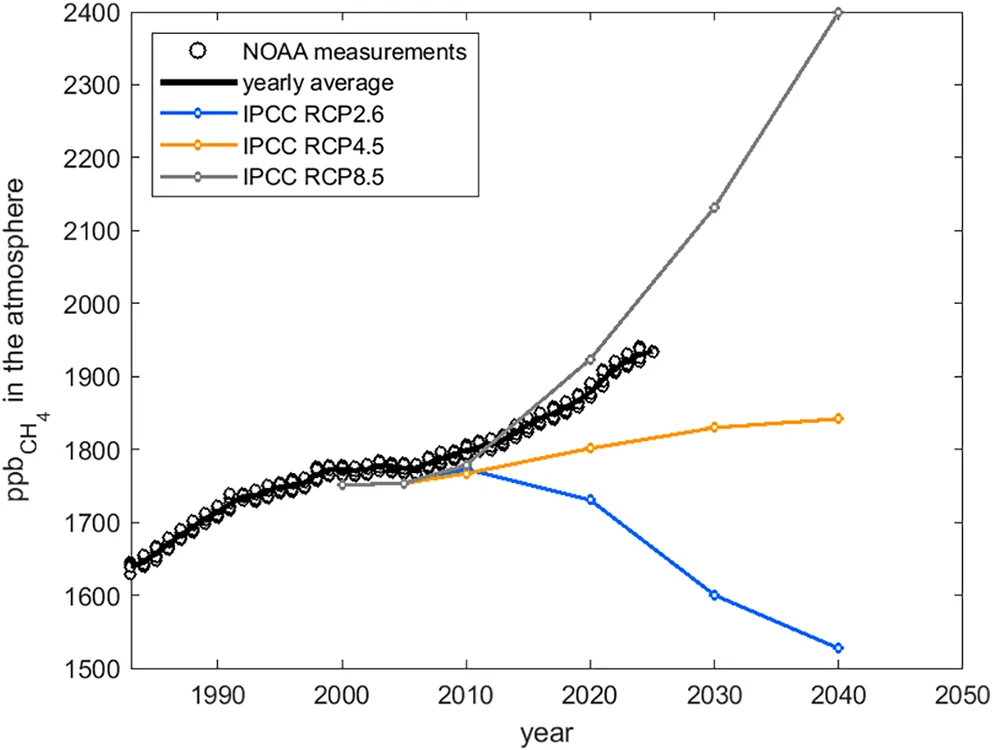
Historical measurements [black dots] and yearly average [black line] of CH_4_ concentration in the atmosphere (in ppb), as reported by NOAA in September 2024;^8^ CH_4_ concentration profiles [gray, orange, and blue line] for different IPCC RCP scenarios.^9^

Methane entering the atmosphere can be curtailed either by fully avoiding its emission, by oxidizing released or ambient methane into CO_2_, or by separating and converting CH_4_ into a final useful product, e.g., plastics. Methane emission reduction via avoidance or point-source oxidation is in many cases the preferred option, often requiring low-cost changes or the implementation of state-of-the-art. For example, the IEA estimates that around 70% of the emissions from the fossil fuel sector could be avoided with existing technologies, such as leak detection and maintenance, at low cost and with a high return.^7^ In contrast to the energy sector, mitigation practices are challenging in the agricultural sector due to the disperse and dilute nature of these emissions; prevention methods can help to partly reduce enteric methane from ruminant animals, but not enough to meet net-zero targets. Accordingly, a substantial gap is likely to exist between methane emission reductions and climate targets, driven primarily by technical constraints on mitigating residual anthropogenic CH_4_ emissions and by projected increases from natural sources.^10^ As recently recognized by both public and private bodies, developing engineered technologies that are capable of removing methane from anthropogenic dilute sources is of paramount importance.^10,11^

To tackle methane emissions from dilute point sources, scientific literature has mostly focused on thermo- and photocatalytic conversion routes. For example, Zhu et al.^12^ suggested a one-step conversion of methane to methanol using a Ni-based catalyst. Krogsbøll et al.^13^ designed a photoreactor for methane abatement through a photochemical system. Sirigina et al.^14^ proposed a promising thermocatalytic process to convert CH_4_ into CO_2_, followed by the capture of the produced CO_2_. While catalytic conversion routes remain promising, a few challenges need to be tackled, including temperature swings in the process and type and amount of catalyst required to process significant volumes of air. However, alternatives to catalytic conversion of methane, among which, most notably, CH_4_ separation, remain largely unexplored.

In fact, adsorption-based processes have proven to be well-suited for capturing CO_2_ from dilute sources; indeed, their “gentle” approach in selective removing target components without needing another intermediate, e.g., water as in amine-based washing, fits particularly well in gas scavenging processes. Not surprisingly, adsorption processes have so far been the preferred avenue in research and development of direct air capture processes, i.e., where CO_2_ is removed from air (430 ppm): solid sorbent separations have been shown to possess relatively higher productivity and lower energy consumption compared to the liquid solvent processes.^15^ Accordingly, adsorption processes have intuitively good potential in separating methane from diluted streams. For example, while nitrogen and methane molecules have similar physicochemical properties, the higher polarizability of the methane allows for a stronger dispersion interaction with solid sorbents, making selective removal possible. Moreover, the low solubility of methane in solvents makes solvent-based separations unsuitable^16^ (in fact, CO_2_ removal from natural gas is carried out with chemical-physical absorption targeting CO_2_ as separated species).

Adsorption is an ever-evolving technique that has long been researched and developed for gas separation, reaching full commercial maturity in several applications, most notably for H_2_ purification and air separation. More recently, the focus of research efforts have been on its application as climate mitigation technology, targeting CO_2_ removal from point sources and from air. The decision to award the Nobel Prize in Chemistry 2025 to researchers who pioneered the development of metal–organic frameworks, a promising class of porous sorbents that can be tailored for specific separations, has once again highlighted the key role adsorption might play in a sustainable society.^17^ An interested reader can easily find several scientific publications focusing on new materials, novel process designs, experimental demonstrations, and techno-economic assessments of adsorption processes. For example, in the context of methane separation by adsorption, numerous studies have focused on upgrading coal mine ventilation air methane (VAM).^18–23^ VAM typically contains 0.2–5 vol % CH_4_. These studies investigate the application of various adsorbent materials, including carbon composites, zeolites, and MOFs, as well as separation processes such as PSA, and VPSA. In this field, the study by Eyer et al.^24^ was seminal in exploring methane removal from air using sorbents: they measured and compared binary N_2_–CH_4_ adsorption isotherms for six different sorbents, including under subambient conditions. However, the scope for methane removal was for analytical application (gas chromatography and eventually isotope measurement) and not as a climate mitigation process. Therefore, to the best of our knowledge, no studies have systematically combined sorbent screening and process design to assess the potential of adsorption for CH_4_ capture from more dilute sources, where CH_4_ is in the range of 2 ppm-1%, such as streams originating from the agricultural sector or directly from ambient air.

The development of any adsorption process starts with the identification of the key ingredient, i.e., the porous sorbent. Today, thanks to advancements in material sciences and computational practices, hundreds of thousands of real *in vitro* and hypothetical *in silico* adsorbents are available.^25^ However, due to the number of possible materials, special care must be taken in devising robust methods that can couple the sorbent choice with process optimization; not surprisingly, this is a particularly computationally demanding task.^26^

The easiest approach to screen adsorption materials relies on the computation of adsorption metrics based on isotherm data, such as the adsorption capacity, selectivity, and heat of adsorption. While this approach offers a useful immediate overview, it cannot give reliable information on the process performance.^27^

Process-based methods are required to achieve a more robust understanding; however, detailed simulation combined with optimization is computationally and time-expensive.^28^ The most time-consuming part of detailed simulations arises from attaining the cyclic steady-state condition (CSS) per simulation and from the number of simulations required to optimize the process conditions. To help with accelerating simulations, machine learning methods can predict CSS conditions as the initial condition of detailed simulations, significantly reducing the computation time.^29^ Alternatively, machine learning can be utilized to predict the process performance from a surrogate model. While the first approach benefits from the accuracy of the detailed model, the computational time is four times that of the surrogate model with respect to the core hours.^30^ On the other hand, in the latter case, the large training data set required to train the models can be promptly computed, but the accuracy of the model is significantly reduced.

Another approach to speed up sorbent screening is to simplify the detailed models by assuming mixed reactor physics at equilibrium, which allows us to substitute a system of partial differential equations solved at CSS conditions with a system of algebraic equations directly solved for equilibrium conditions.^31^ When the bed is also considered saturated (or at a predefined saturation level) at the end of the adsorption step, the system of equations is further simplified by knowing the gas uptake at the start of regeneration.

Despite the rather strong assumption of well-mixed equilibrium conditions, such models have proven to perform reasonably well compared to rate-based models, especially when used as a first step to understand process and material coupling. Therefore, equilibrium adsorption models have recently been used in several studies, including process and material screening for different cycle types. For example, Maring and Webley applied this approach to CO_2_ capture from flue gas using vacuum swing adsorption (VSA) to evaluate the operating conditions and provide suggestions for material development.^31^ Joss et al. developed a simplified model for the temperature swing adsorption (TSA) process to capture CO_2_ from the flue gases^32^ where the heat transfer kinetics is retained, and a semianalytical solution of the CCS condition is computed. Building on this framework, Ajenifuja et al. have later proposed a new shortcut model for high-throughput material screening in TSA processes.^33^ Similarly, Balashankar et al. used an isothermal equilibrium model to screen the NIST/ARPA-E database in order to identify promising adsorbents for postcombustion CO_2_ capture applications with a VSA process.^34^ Wang et al.^35^ have implemented an equilibrium model for pressure–temperature swing adsorption (PTSA) and validated it with a 2D detailed model; the model was used to screen 20 materials for carbon capture from flue gases. More recently, Grimm and Gazzani^36^ have utilized machine learning to aid equilibrium models in computing the cycle productivity; the model was employed to identify promising sorbents for CO_2_ capture from dilute sources (400 ppm-1 vol %) using a four-step vacuum temperature swing adsorption (VTSA) process.

While equilibrium models are effective tools at an early development stage to better understand the process key features and identify suitable sorbents, it is important to stress that they do not substitute more detailed 1- or 2-D rate-based models. Rather, they allow one to develop intuition for the process before moving to a more detailed simulation approach.

In this work, we provide the first systematic process-level assessment of adsorption for methane removal from ultradilute streams. By combining process conceptualization, large-scale sorbent screening, and process modeling, we evaluate whether ppm-level CH_4_ separation from agriculture- and air-like mixtures is thermodynamically feasible. The primary objective is to identify promising sorbents through process-based modeling and optimization, while explicitly accounting for the simplifications inherent to equilibrium approaches. The key contributions of this work are

- We assess the feasibility of adsorption processes for methane removal from ultradilute streams, considering CH_4_ concentrations representative of atmospheric and agricultural conditions (100, 25, and 2 ppm).
- We propose and analyze a process concept for methane capture by adsorption from ultradilute streams.
- We develop a process-based computational screening framework for sorbent evaluation, extending the approach of Grimm et al.^36^ to methane removal and applying it to more than 8000 candidate materials from the NIST database.
- We consider ternary CH_4_–CO_2_–N_2_ mixtures, highlighting critical gaps in adsorption data for key components of the separation and showing the impact of using unary or competitive isotherm models for selected sorbents.
- We account for N_2_ as an adsorbing component rather than treating it as inert, showing that despite its weak adsorption, its large molar fraction significantly influences sorbent ranking and process performance.^37^
- We compare the equilibrium-based screening framework to an established one-dimensional rate-based adsorption model.

The rest of the paper is organized as follows. In the methodology section, we present the zero-dimensional (0D) model and the overall screening method, including the process optimization; moreover, we describe the adsorption process. In the results, we first validate the 0D model against a detailed one-dimensional (1D) model and proceed to the application of the model to the sorbent screening. Finally, we discuss the limits, possible improvements, and we draw conclusions.

## Methodology

Given the novelty of the application investigated and the lack of experimental data required for rate-based modeling, in this work we develop and use an equilibrium model tuned for methane removal. More specifically, the model builds upon the framework described in Grimm and Gazzani^36^ and includes the following modifications tailored toward methane capture:

- The gas phase consists of a ternary CO_2_–CH_4_–N_2_ mixture (instead of a binary CO_2_–N_2_ mixture with N_2_ treated as inert).
- Subambient temperature operation is added, with the adsorption temperature now treated as an optimization variable (instead of constant, ambient temperature).
- The model does not compute productivity due to the lack of 1-D training data in this phase.
- The process optimization algorithm was updated to use a more computationally efficient approach.

### Equilibrium Model

The adsorption cycle considered in this work, which is depicted in Figure 2 for air as feed, is a VTSA process similar to DAC application, and consists of 4 stages: adsorption, vacuum blow-down for bed cleaning, open heating at vacuum conditions, and closed cooling with repressurization. The model considers three-component mixtures of CH_4_, CO_2_, and N_2_. During the adsorption step, all three species can adsorb according to equilibrium. In the subsequent vacuum blow-down step, desorption starts, with nitrogen desorbing preferentially over CH_4_ and CO_2_; this allows to clean the bed before collecting the product during heating, limiting the amount of N_2_ in the product. Heating is applied to desorb the bulk of the remaining adsorbed molecules. Finally, cooling and repressurization bring the bed to adsorption conditions. The equilibrium model relies on the following assumptions:

- The bed is a well-mixed reactor with no radial or longitudinal concentration gradients.
- The solid and gas phases are always in thermal and chemical equilibrium, with negligible heat and mass transfer resistances.
- The gas phase behaves like an ideal gas with a constant heat capacity.
- The pressure drop in the bed is considered as negligible.
- The specific heat capacity of the gas phase is considered negligible.

**Figure 2:**
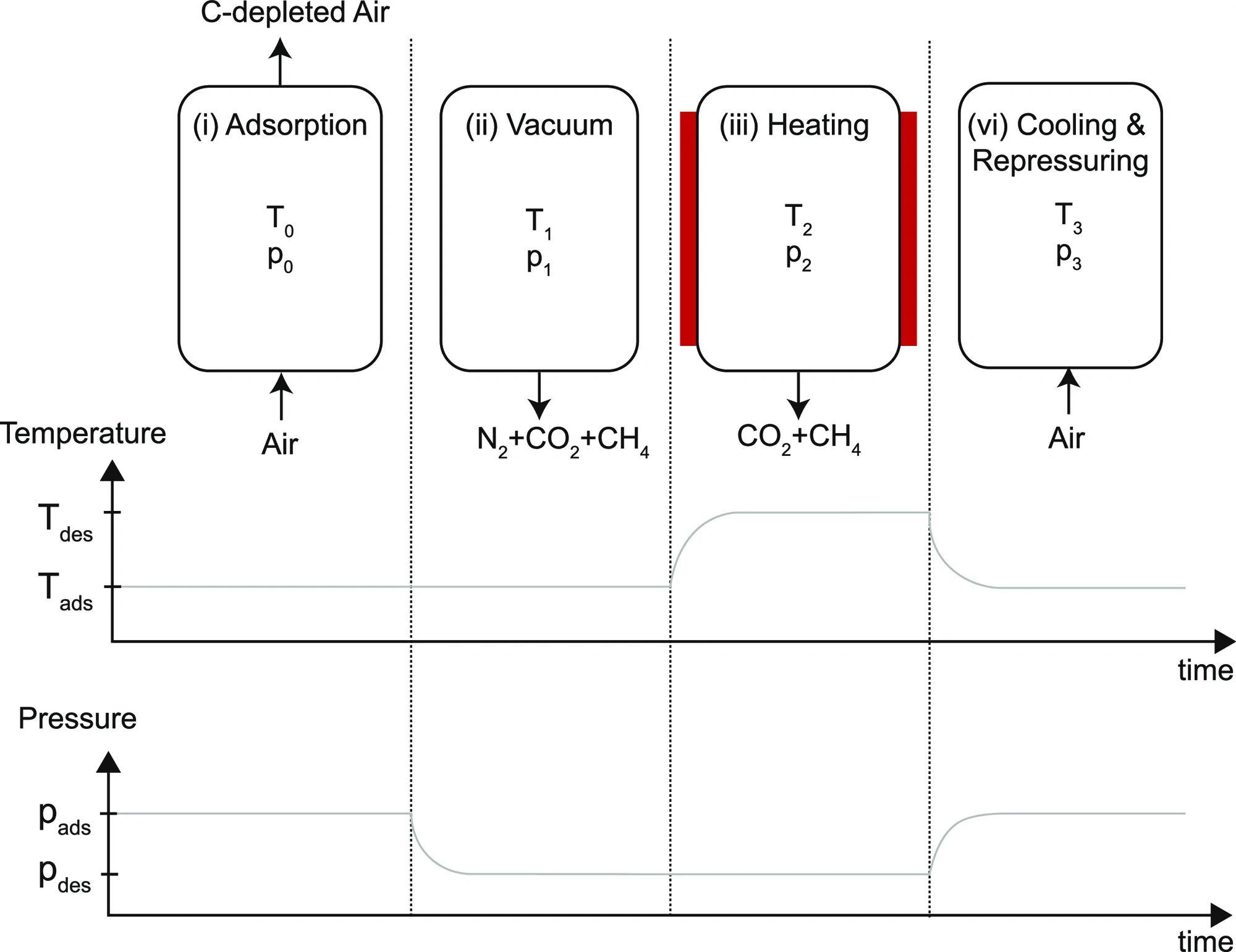
Process scheme of the four-step VTSA cycle. Top: cycle steps including adsorption, vacumm, heating, and cooling & repressurization. Bottom: qualitative temperature and pressure profiles throughout the cycle.

Accordingly, the overall material balance considering the column as the system boundary can be written as

1 $$\sum_{i}N_{i,\mathrm{total}}\left(t_{f}\right)-\sum_{i}N_{i,\mathrm{total}}\left(t_{0}\right)=\int_{t_{0}}^{t_{f}}{Ṅ}_{\mathrm{in}}dt-\int_{t_{0}}^{t_{f}}{Ṅ}_{\mathrm{out}}dt$$

In the eq 1, *Ṅ*_in_ is the total molar flow rate entering the column, *Ṅ*_out_ is the total molar flow rate exiting the column, and *N*_*i*,total_ is the total number of moles of component *i* in the bed, which is given by the sum of the moles in the solid (s) and in the fluid (f) phase:

2 $$N_{i,\mathrm{total}}\left(t\right)=N_{i,s}\left(t\right)+N_{i,f}\left(t\right)$$

Furthermore, the total number of moles in the solid phase is described by a gas isotherm model:

3 $$N_{i,s}\left(t\right)=m_{s}q_{i}^{*}\left(y_{i},p,T\right)$$

where *m*_s_ is the mass of the adsorbent and *q*_i_^*^ is the equilibrium adsorbed amount.

The equilibrium adsorbed amount can be calculated from a suitable isotherm model, generally described by *q*_i_^*^ = *f*(*y*_*i*_, *p*, *T*), where *y*_*i*_ is the mole fraction of component *i*, *p* is the pressure in the column, and *T* is the temperature. Depending on the sorbent behavior, the Toth, Langmuir–Freundlich, or S-shaped isotherm model can be used to calculate the equilibrium adsorbed amount. Further details about the isotherm equations can be found in Section S1.

The fluid phase in eq 2 is described by the ideal gas law:

4 $$N_{i,f}\left(t\right)=\left(py_{i}V_{c}\epsilon \right)/\mathrm{RT}$$

where *V*_c_ is the column volume, ϵ the bed void fraction and *R* represents the universal gas constant. The overall energy balance, after neglecting the specific heat capacity of the fluid phase, can be written as

5 $$m_{s}c_{p,s}\left(T\left(t_{f}\right)-T\left(t_{0}\right)\right)=\int_{t_{0}}^{t_{f}}\mathrm{Q̇d}t+\left(N_{i,s}\left(t_{f}\right)-N_{i,s}\left(t_{0}\right)\right)\sum_{i}^{n}\left|\Delta H_{\mathrm{ads},i}\right|$$

where *Q̇* is the thermal energy during heating or cooling (positive during heating). The isosteric heat of adsorption can be calculated from the Clausius–Clapeyron equation:

6 $$\left(\frac{\partial \ln p_{i}}{\partial T}\right)=\frac{-\Delta H_{\mathrm{ads},i}}{RT^{2}}$$

Table S2 summarizes the material and energy balance equations derived for each step of the VTSA process. It is important to note that the starting point for the resolution of the equilibrium model is the end of adsorption step, i.e., where the bed is assumed to be saturated with respect to the most adsorbing species; accordingly, the model starts by solving the blow-down step of the cycle. The model is implemented in Matlab r2022b, where the resulting system of algebraic equations is solved numerically using *Lsqnonlin*. For the simulation of the cycle, the model requires having the final value of temperature and pressure of each cycle step; these values are decided using the optimization algorithm described below. Moreover, and exclusively for the purpose of the model verification, temperature and pressure profiles are assigned to compare the outlet composition over time with the results of the 1D model; no profiles are used in the sorbent screening.

### Process Concept and Performance Indicators

The overall process that we consider for removing methane from dilute sources is shown in Figure 3. Due to the similar behavior of N_2_ and CH_4_, we have designed a process where adsorption can be carried out, if necessary, at subambient temperature. Under these conditions, sorbents can be more selective toward CH_4_ than N_2_.^24^ In order to do that efficiently while also exploiting the subambient conditions, which require and favor water removal, heat integration is included in the process. Here, we follow the process proposed in the patent by Tor Christensen,^38^ which was designed for CO_2_ capture from air using hydrophilic physical sorbents, e.g., Zeolite 13X. This process makes use of concepts already applied in cryogenic gas applications, such as liquefied natural gas and air separation units. First, a combined dryer-heat exchanger is used to cool the incoming stream from *T*_amb_ to *T*_1_ while also removing water by condensation and adsorption (for example, an enthalpy wheel coated with H_2_O sorbents, as proposed in ref 38). The cooling medium, which also regenerates the drying unit thanks to its dry condition, is the cold carbon-depleted air leaving the CH_4_ capture unit. A second heat exchanger cools the incoming stream to the operating temperature of the VTSA cycle by using an external cooling machine. Minimum temperature differences (Δ*T*_min_) are considered for the two heat exchangers, 5 °C in the refrigeration heat exchanger (gas-evaporating fluid) and 10 °C in the regenerative heat exchanger (gas–gas). We note that at these conditions—subambient temperature and ambient pressure after a drying bed - the absolute water content in the inlet stream can be considered zero. The drying unit itself is not explicitly simulated in this work, as it it is considered a noncritical unit, especially if compared to the CH_4_ removal step. Accordingly, this process configuration allows us to also use hydrophilic materials, such as zeolite 13X, while exploiting CH_4_ selectivity at low temperatures.

**Figure 3:**
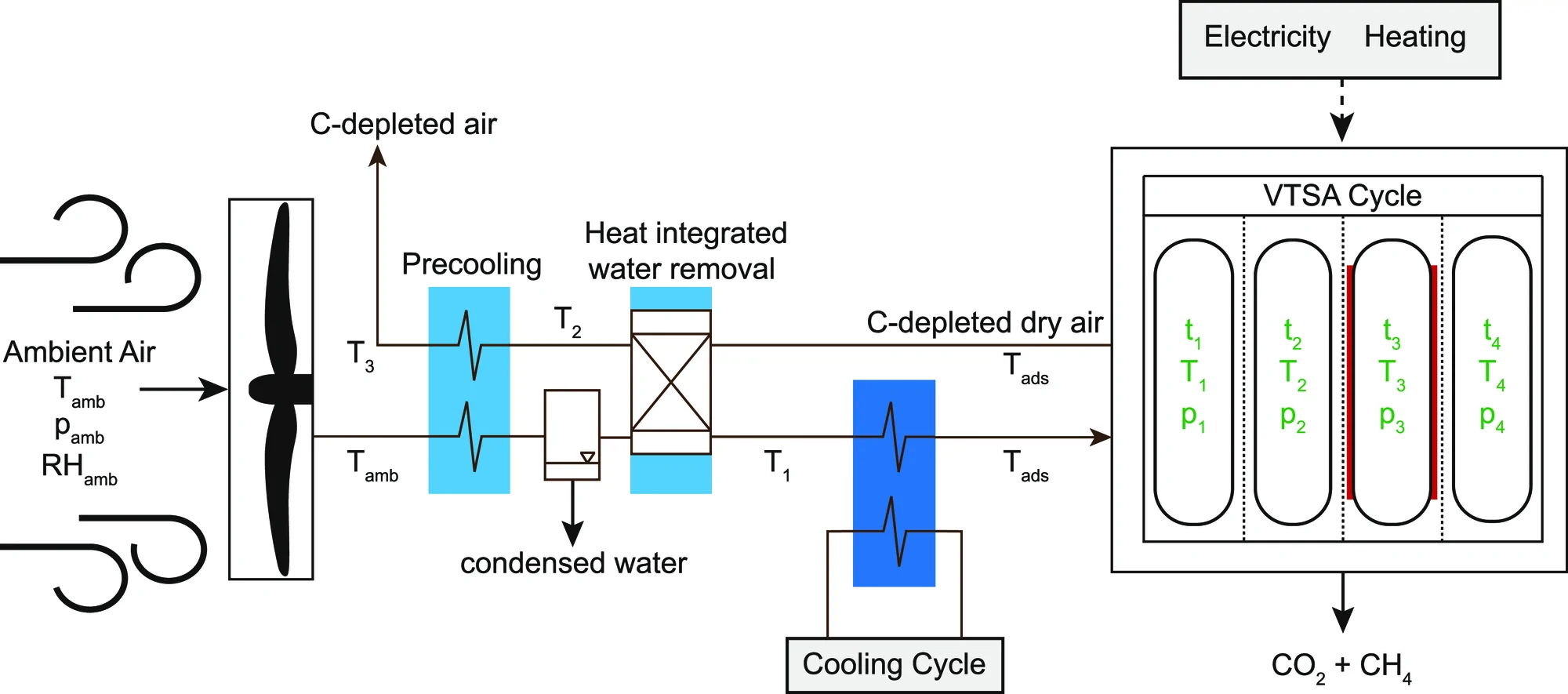
Overall methane removal process. Starting from the inlet (left to right panels): blower, heat integration for precooling and cooling, water removal, adsorption cycle.

We evaluate the process using different key indicators, including the separation and energy performance.

The purity *Pur*_*i*_ and the recovery *Rec*_*i*_ are calculated as

7 $$\mathrm{Pu}r_{i}=\frac{N_{i}}{N_{\mathrm{tot}}^{\mathrm{out}}}$$

8 Reci=NiNiin

where *N*_*i*_ is the number of moles of component *i* leaving the bed during the vacuum and heating steps, *N*_out_^tot^ is the total number of moles leaving the bed during the vacuum and heating steps, *N*_in_^in^ is the number of moles of component *i* entering the bed, and *i* can refer to either CH_4_, CO_2_, or CO_2_eq depending on the targeted product, which in the following is considered to be CH_4_.

Given the diverse energy input, namely, electricity and heat, we evaluate the energy performance using the specific exergy consumption, calculated as

9 $$B_{i}=\frac{B_{\mathrm{tot}}}{m_{i}}$$

where *B*_*i*_ is the specific mass exergy consumption, *B*_tot_ the total exergy consumption, and *m*_*i*_ is the mass of the desired product leaving the bed during the vacuum and heating steps. When computing this value for methane, we adjust the specific exergy consumption in terms of CO_2_ equivalent, i.e., the mass of CH_4_ removed is multiplied by the global warming potential (GWP) on a 100 year basis. In such case, *GWP*_CH_4__ = 28; this value can be used to recompute the energy per kg of methane removed. Using the CO_2_ equivalent framework allows us to value both methane and CO_2_ in the product, while also positioning the separation with respect to other separations (e.g., DAC). At the same time, we recognize that—given the complexity of CH_4_ role on climate warming and climate mitigation—the CO_2_ equivalent framework does not allow for a full evaluation of the separation technology in the context of climate change; however, more complex KPIs would need different models (e.g., integrated assessment models) which are not in the scope of this work. The total exergy consumption is given by three processes: heating, cooling, and vacuum pump (in the following *B* is used to refer to exergy).

10 $$B_{\mathrm{tot}}=B_{\mathrm{heat}}+B_{\mathrm{cool}}+B_{\mathrm{vac}}$$

*B*_heat_ is calculated using the thermal energy demand of the process and the Carnot factor:

11 $$B_{\mathrm{heat}}=Q_{\mathrm{th},\mathrm{heat}}\left(1-\frac{T_{\mathrm{amb}}}{T_{\mathrm{des}}}\right)$$

where *T*_amb_ is the ambient temperature and *T*_des_ is the maximum temperature during the desorption step.

In order to calculate the electricity demand of the cooling cycle, which also corresponds to *B*_cool_, we use the energy balance of the heat integration process and consider a second law thermodynamic efficiency of the heat pump of 70%. The calculation of the energy balance of the cooling cycle is further explained in the Section S2.

12 $$T_{1}=T_{\mathrm{amb}}+T_{\mathrm{ads}}-T_{3}$$

13 $$Q_{\mathrm{th},\mathrm{cool}}=N_{\mathrm{in}}C_{p}\left(T_{1}-T_{\mathrm{ads}}\right)$$

14 $$B_{\mathrm{cool}}=\frac{Q_{\mathrm{th},\mathrm{cool}}}{\eta_{\mathrm{II}‐\mathrm{law}}{\mathrm{COP}}_{\mathrm{Carnot}}}$$

Finally, the electricity consumption of the vacuum pump, which corresponds to *B*_vac_ can be computed as

15 $$B_{\mathrm{vac}}=\frac{1}{\eta }Ṅ\left(\frac{k}{k-1}\right)\mathrm{RT}\left({\left(\frac{p_{\mathrm{out}}}{p_{\mathrm{in}}}\right)}^{\left(k-1/k\right)}-1\right)$$

16 $$k=\frac{c_{p}}{c_{v}}$$

### Process Optimization

Adsorption cycles are complex due to the inherent dynamic behavior and the interplay between several control variables, such as temperature levels, cycle times, flow rates. While equilibrium models remove the time domain, several variables remain to be fixed to compute the process performance. To this end, it is important to identify optimal operating points via rigorous mathematical optimization. In this work, we do that by coupling the equilibrium model with a recent, high performing multiobjective optimization algorithm: the Thompson sampling method^39^ (TSEMO, as implemented in Matlab R2018a^40^). More specifically, we consider as objective functions (i) the purity of methane (maximized) and (ii) the specific exergy consumption (minimized). The decision variables of the optimization are adsorption temperature *T*_ads_, desorption temperature *T*_des_ and vacuum pressure level *p*_vac_. The values respective values are shown in Table 1. The following rationale is behind the bounds: (i) the lower bound of the adsorption temperature is fixed to enable subambient operation while keeping the process simple enough for distributed applications such as farming (−20 °C can be achieved with regular households cooling cycles); (ii) the maximum desorption temperature is limited to 100.0 °C to prevent—in a first approximation - material degradation; (iii) the minimum vacuum pressure is (optimiztically) fixed to 0.01 bar. Among these bounds, the low vacuum pressure is the most technologically challenging, while the other two do not present particular concerns.

**Table 1:** Process Optimization Decision Variables and Their Lower and Upper Boundaries

*T*_ads_ (*K*)	*T*_des_ (*K*)	*p*_vac_ (*bar*)
253.15–293.15	303.15–373.15	0.01–0.8

## Results: Model Verification

The verification of the new equilibrium VTSA model is conducted by comparing the results with a more detailed 1D rate-based model. The detailed one-dimensional model solves numerically the partial differential equations (PDEs) arising from mass and energy balances, until a cyclic steady state (CSS) solution is found. The 1D model employed for this verification has been discussed in several scientific publications, including model validation against experimental results.^41–43^ A short description of the 1D model is provided in Section S3; the interested reader can find additional details in the scientific literature cited above.

For verification purposes, the input conditions for the 0D and 1D models must align. Here, we consider as feed a ternary air mixture with 1800 ppm of CO_2_, 500 ppm of CH_4_, and the rest N_2_. The temperature and pressure profiles, which are required inputs for the 0D model, are directly taken from the 1D model. As for the adsorbents, two materials are considered in the validation: (i) activated carbon A and (ii) ZIF69. The Toth single gas isotherm model is used to compute equilibrium adsorption for both sorbents.

As noted above, time is the critical variable absent from equilibrium models when compared with rate-based models. To enable comparison of adsorption profiles, which are time-dependent in rate-based models, the adsorption step in the equilibrium model is divided into multiple substeps (see Grimm and Gazzani^36^ for more details). Moreover, in the 1D model, the durations of the blowdown (*t*_BD_), heating (*t*_heating_), and cooling (*t*_cooling_) steps are defined such that the desired final temperature and pressure are achieved; these parameters are instead specified as inputs in the 0D models. Likewise, the adsorption time (*t*_ads_) in the 1D model is sufficiently long to ensure a complete bed saturation.

Figure 4 compares the molar fractions of the 0D and the 1D models at the column outlet for activated carbon A and ZIF69 (note that for the 0D model the concentration profile is uniform throughout the adsorption bed, while for the 1D model we consider the outlet in the direction of the flow). The comparison of the profiles shows that the models are in good agreement despite the simplicity of the 0D model (here, it is important to remember that equilibrium adsorption models cannot fully reproduce rate-based, 1D adsorption models).

**Figure 4:**
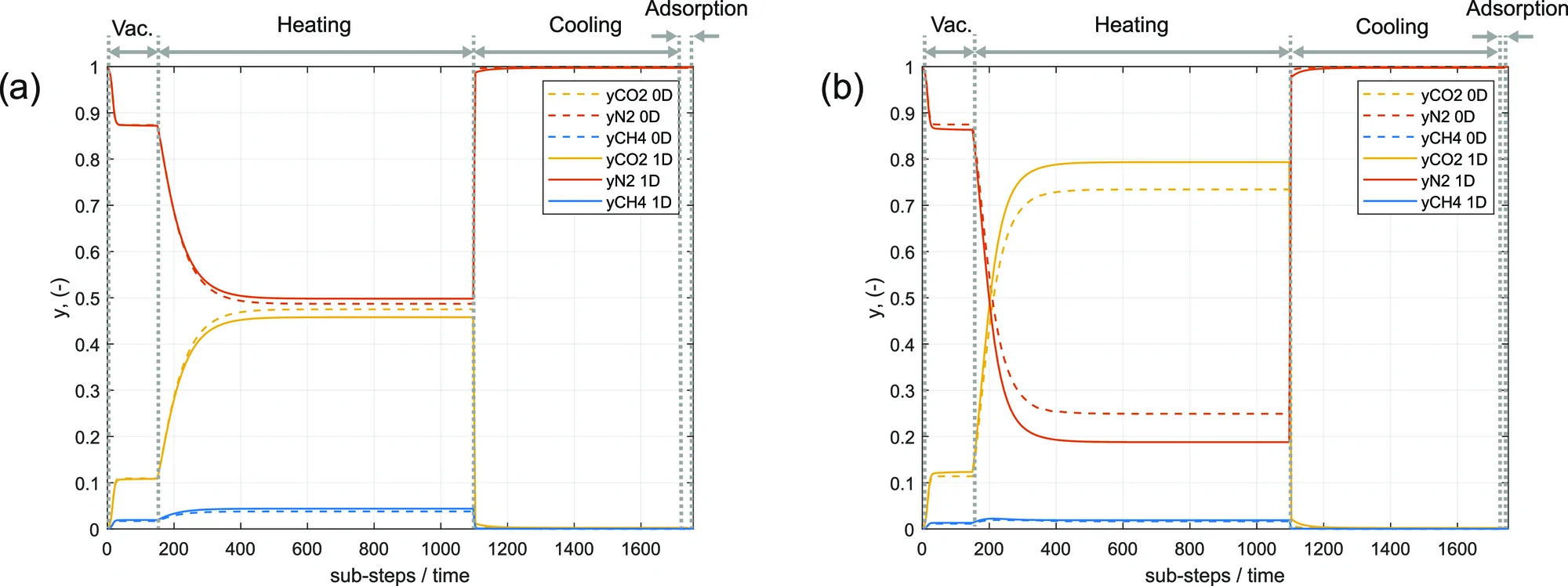
Concentration profiles along an exemplary cycle for the 0D and 1D models (at the column outlet): (a) Activated Carbon A; (b) ZIF69. The 0D model profiles are computed as substeps while the 1D model profiles are varying in time.

To reveal the interplay between different model variables, Figure 5 compares the cycle profiles for the 0D and 1D model in terms of molar fraction-temperature and molar fraction-pressure (for activated carbon A, while for ZIF69 the profiles can be found in the Section S4). Within the scope of the model verification, the cycle profiles are in good agreement with some relevant deviations as expected when introducing the equilibrium simplification. As shown in the graphs, the largest deviation arises during the cooling step, which has a large gradient in space in the 1D model, while the concentration profile is assumed to be fully mixed in the 0D model. During the adsorption step, the molar fraction-temperature/pressure profiles are in good agreement, suggesting that the 0D model can also be used as a tool to predict the concentration evolution during an adsorption cycle.

**Figure 5:**
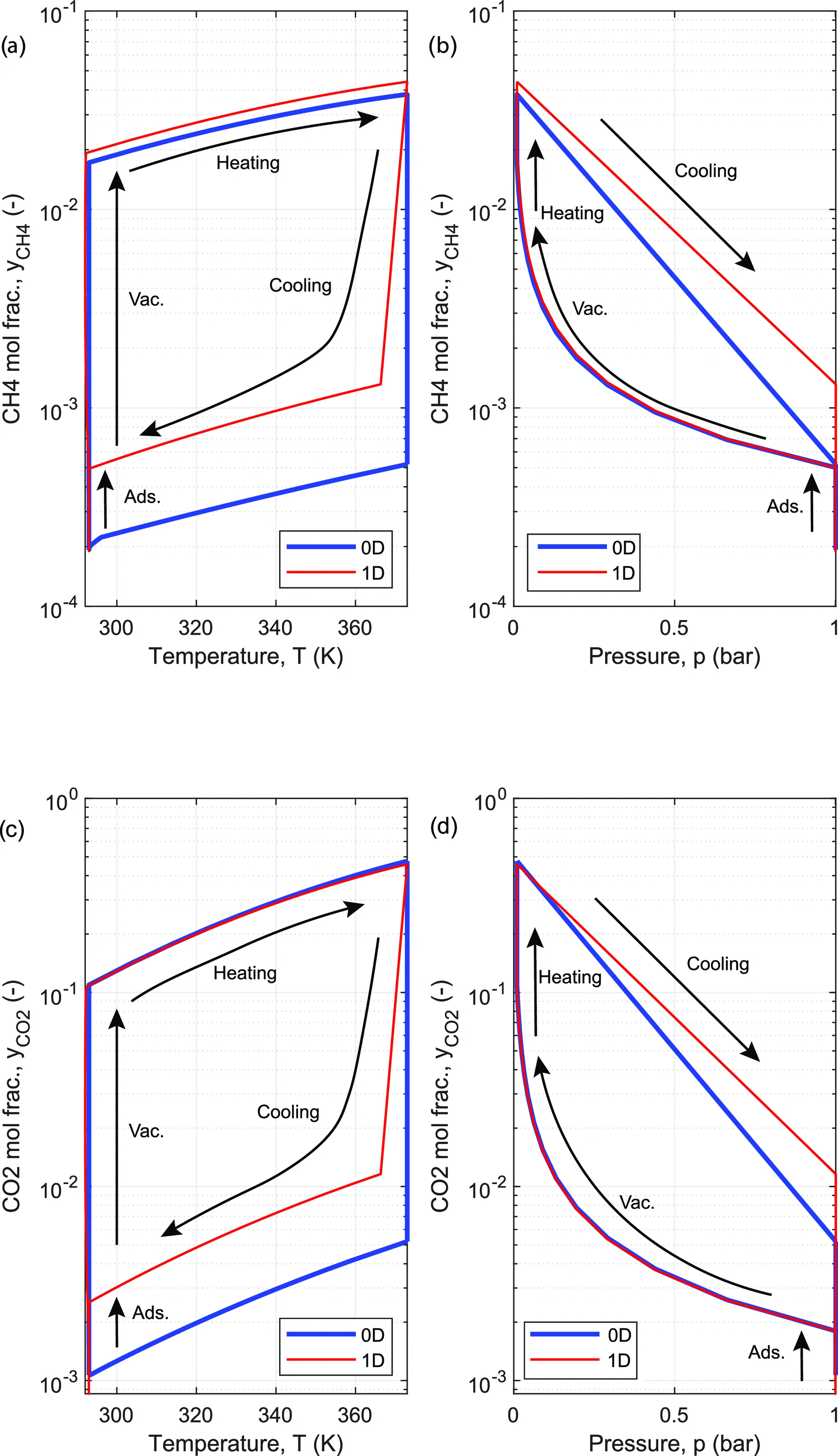
Cycle profiles comparison for the case of activated carbon A: Top panel: Molar fraction of CH_4_ vs temperature (a) and pressure (b). Bottom panel: Molar fraction of CO_2_ vs temperature (c) and pressure (d). For the 1D model, the temperature, pressure, and mol frac. plots refer to the values at the bed outlet considering cyclic steady sate conditions.

To further compare the 0D and 1D models, the process performance of the VTSA cycle was computed for a combination of different vacuum pressures and heating temperatures for each material. The investigated conditions are presented in Table 2. Both models are executed considering the same set of conditions; moreover, the temperature and pressure profiles of the 1D model are transferred as inputs to the 0D model. Additional details for the 1D and 0D simulations parameters can be found in Table S4. Figure 6 shows the resulting parity plots of the process performances, namely, purity, recovery, cyclic working capacity, and specific exergy consumption. The process performance is calculated for CH_4_, except for the specific exergy consumption, which is computed based on CO_2eq_. The process performance for CO_2_ is shown in Figure S2.

**Figure 6:**
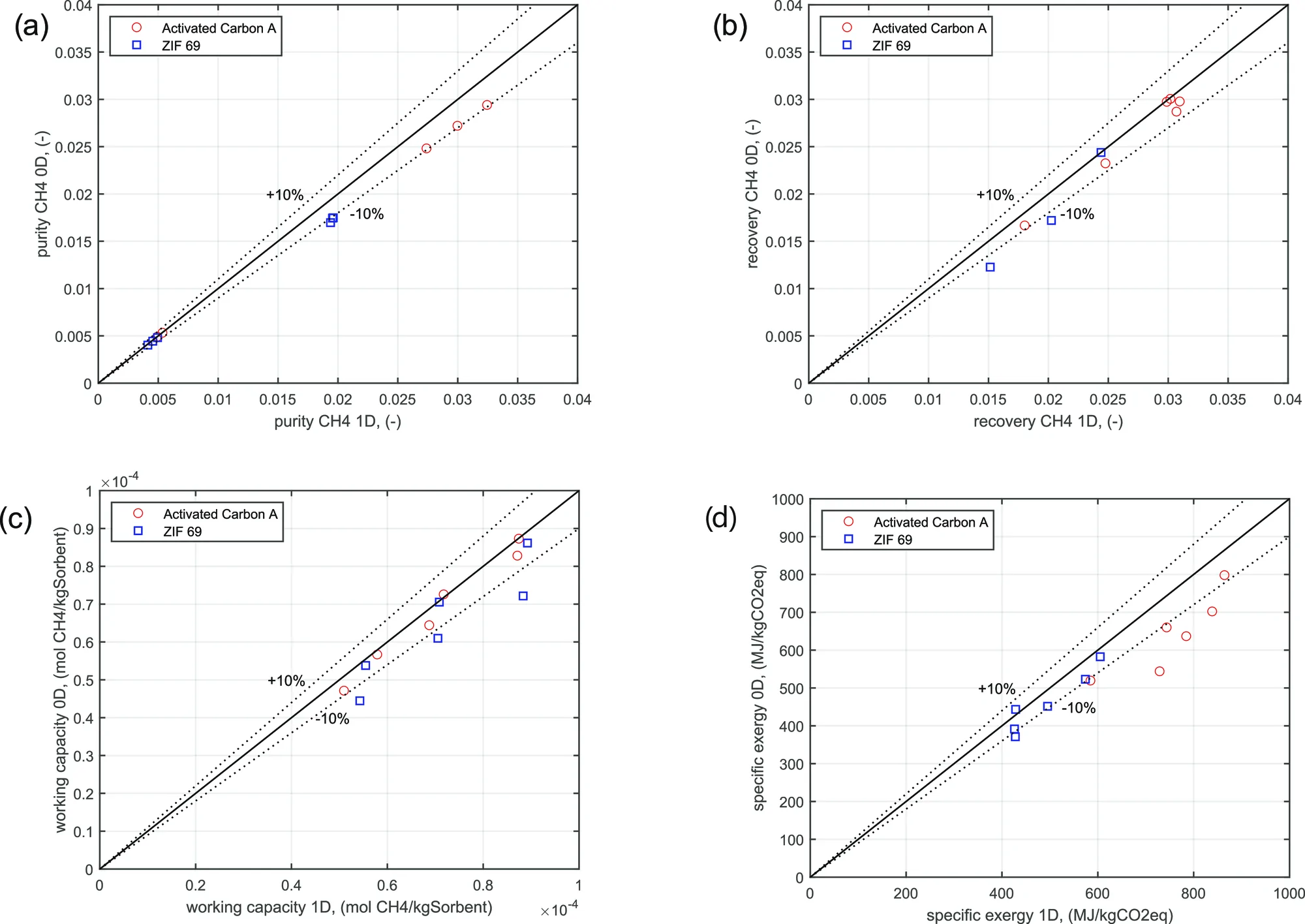
Parity plots of the performance parameters of the 0D model (*y*-axis) and the 1D model (*x*-axis). Results for activated carbon A are shown with red empty dots. Results for ZIF69 are shown with blue empty squares. The dashed lines show a margin of 10%.

**Table 2:** Process Conditions Considered for Performance Comparison of the 0D Model against the 1D Model

# run	*p*_vac_ (bar)	*T*_des_ (K)	# run	*p*_vac_ (bar)	*T*_des_ (K)
1	0.1	253	4	0.01	253
2	0.1	373	5	0.01	273
3	0.1	393	6	0.01	293

As shown in the parity plots, the process performances predicted by the 0D and 1D models are in good agreement with most of the results falling within the 10% gap. In the separation performance (purity and recovery), only two out of 24 results are slightly outside the 10% gap, both cases belonging to ZIF 69. For the working capacity, three points out of 12 (again ZIF 69) have under-predicted results. Notably, while the separation performance is in very good agreement, the specific exergy consumption is consistently under-predicted by the 0D model for all but one condition, with three points being significantly below the 10% gap. This behavior, which is common in 0D models, can be explained by the double effect of recovery (numerator) and energy consumption (denominator). The latter is particularly prone to deviations due to the simplified (isothermal) energy balance and the assumption of a constant heat of adsorption.^36^

In light of these results, we can conclude that the 0D model provides a sufficiently precise, fast, and simple platform for large-scale sorbent screening applied to methane removal. It should, however, be noted that the developed equilibrium model does not substitute the rate-base models during a more detailed process design phase: after promising sorbents are identified and preliminary process understanding developed, further investigations with rate-based modeling are necessary to obtain more accurate insights.

## Results: Sorbents Screening

The equilibrium model and computing framework described above are used to screen the NIST/ARPA-E database,^44^ which contains isotherms for >8000 materials, in order to identify potential materials for methane capture. We apply the screening method to three different types of diluted non-fossil sources, ranging from conditions found in dairy stables and directly from the air. Since the concentration of methane in dairy stables depends highly on the type of construction, we consider both an open and a semiclosed structure. In the latter, methane concentration can reach up to 175 ppm during winter, where walls are closed with linen-type and ventilation air is maintained at a low level. Here, we base the composition of the inlet mixtures from barns on the direct measurements carried out in the EU project CANMILK.^45^ It is important to note that CO_2_ is another important greenhouse gas present in significant amounts in stables, and its concentration varies along with that of methane. As CO_2_ can be a strong adsorptive component, we carry out the screening for ternary mixtures of CH_4_–CO_2_–N_2_. Table 3 summarizes the case studies investigated in this work.

**Table 3:** Diluted CH_4_ Streams Considered for the Sorbent Screening, with Nitrogen Added to Reach Unity

mixture	CH_4_ concentration (ppm)	CO_2_ concentration (ppm)
1. Semiclosed stables	100	1200
2. Open stables	25	700
3. Air	2	400

Due to the extent and complexity of the data reported in the NIST/ARPA-E database, special care must be taken to ensure that only relevant materials are considered for the process-based screening. Therefore, several steps as required, which are also depicted in Figure 7. As first step, the received data is sorted to check the availability for the desired gas isotherms and to uniform the units; notably, materials with less than two adsorption temperatures are excluded. Then, the isotherm fitting is executed using the Toth, Langmuir Freundlich, and S-shaped models (the most precise model is used in the next steps). A check on the working capacity is carried out in this phase, where materials with negative working capacity at the most convenient set of operating variables are disregarded. Retrieving data, sorting and fitting is done separately for CH_4_, CO_2_ and N_2_ and the resulting parameters are given as input to the 0D model. However, when no data for N_2_ or CO_2_ adsorption is available, adsorption behavior of MIL-101 ^46^ is considered in the model (this is a rather conservative approach with respect to methane, given the good capacity of MIL-101 for CO_2_). Moreover, in case of lack of physical properties, including material density and porosity, these generic assumptions are made: ρ_material_ = 1130 kg/m^3^, particle void fraction (ϵ_particle_) = 0.35, and *C*_p,s_ = 1070 J (kg/K). Although physical properties are important and must eventually considered precisely for a robust and detailed process design, these properties are unlikely to have substantial effect on the material ranking results. After an initial process simulation with the 0D model, materials with unreasonable process performance are left out from further considerations, while the remaining data set is considered for process optimization with the 0D model.

**Figure 7:**
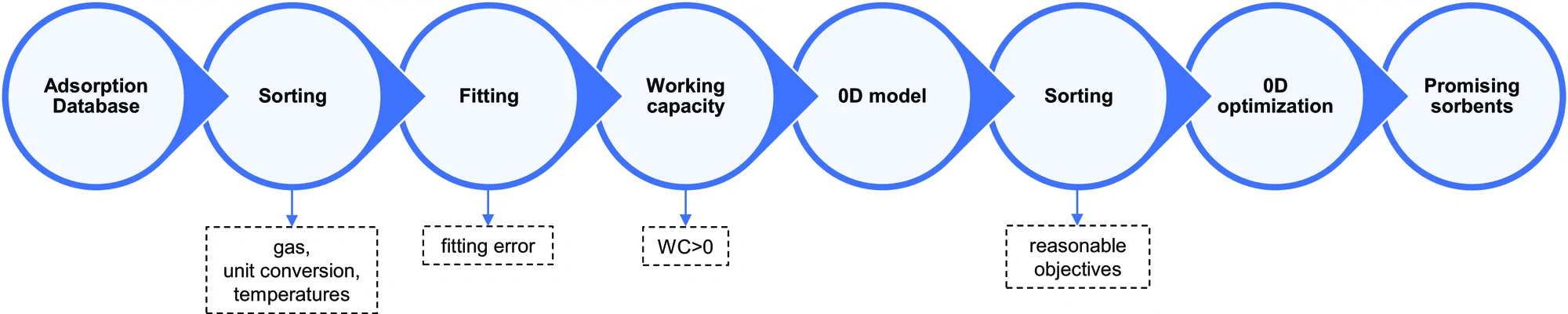
Steps of screening methodology.

### Sorbents Screening

Starting from the 8000 materials in the database, 365 materials were fitted with CO_2_ isotherms, 178 with CH_4_ isotherms, and 70 with N_2_ isotherms. The 178 materials with CH_4_ isotherms were used as input to the 0D simulation.

We show the results of the screening in Figure 8 by using CH_4_–CO_2_–N_2_ ternary diagrams of the product composition (molar fraction), where we color code the sorbent symbol according to the specific exergy consumption. The precise numerical results are shown in Table 4, while the associated optimal operating conditions are shown in Table S5.

**Figure 8:**
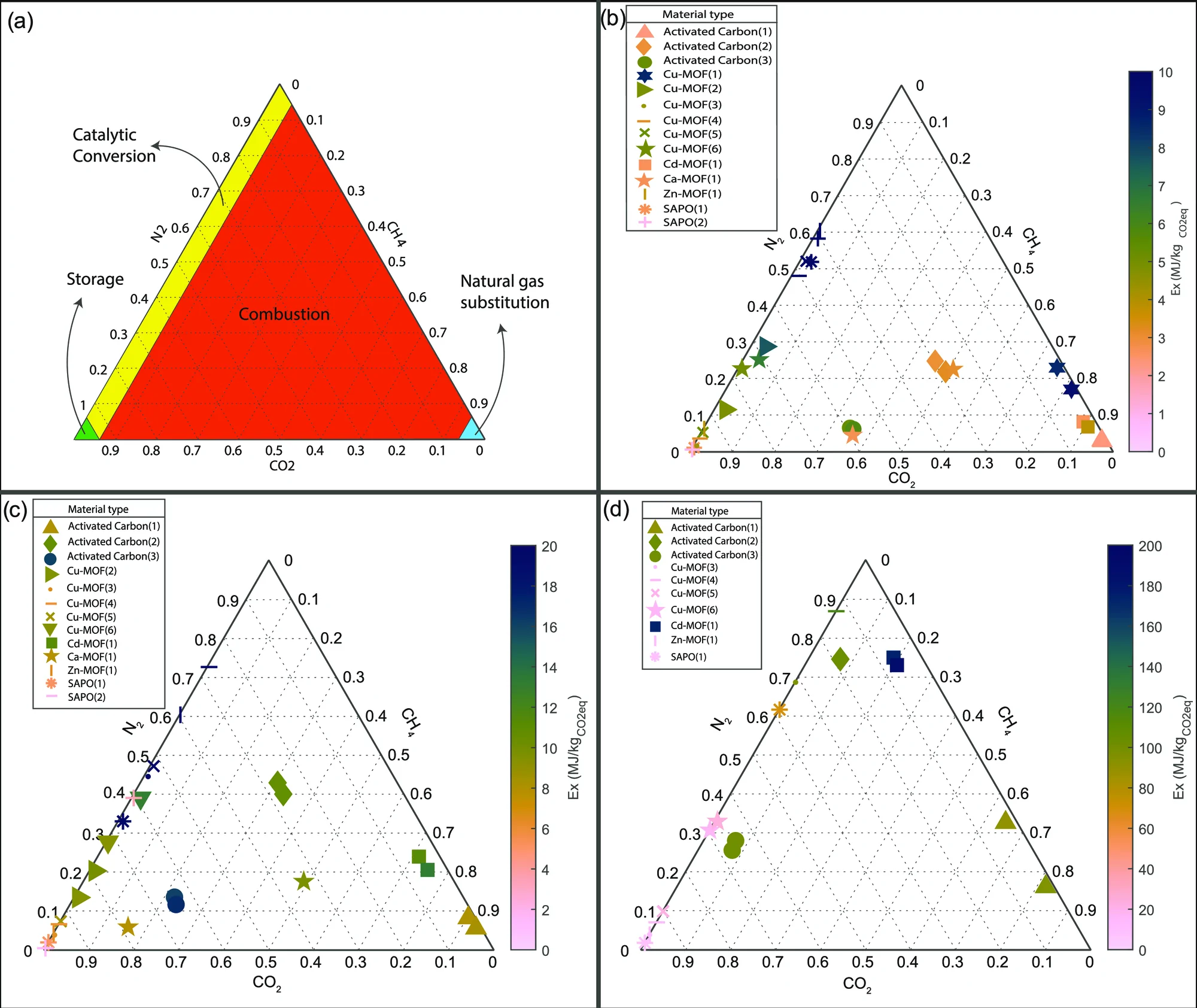
Ternary diagram of product molar fractions; different symbols represent different sorbents and are colored according to the exergy consumption. (a) Overview of possible downstream applications. (b–d) Results for the different feed conditions. (b) 100 ppm of CH_4_-1200 ppm of CO_2_; (c) 25 ppm of CH_4_-700 ppm of CO_2_; and (d) 2 ppm of CH_4_-400 ppm of CO_2_. Materials: Activated carbon(1),^47^ activated carbon(2),^48^ activated carbon(3),^49^ Cu-MOF(1),^50^ Cu-MOF(2),^51^ Cu-MOF(3),^52^ Cu-MOF(4),^53^ Cu-MOF(5),^54^ Cu-MOF(6),^55^ Cd-MOF(1),^56^ Ca-MOF(1),^57^ Zn-MOF(1),^54^ SAPO(1),^58^ and SAPO(2).^59^ The color range of the specific exergy consumption of the capture process is adjusted per case to highlight the different performance of the materials.

**Table 4:** Separation Performance and Exergy Consumption for the Different Feedstock Conditions^a^

	100 ppm of CH_4_-1200 ppm of CO_2_			25 ppm of CH_4_-700 ppm of CO_2_			2 ppm of CH_4_-400 ppm of CO_2_		
material name	purity CH_4_ (%)	purity CO_2_ (%)	specific exergy (MJ/kg CO_2_eq)	purity CH_4_ (%)	purity CO_2_ (%)	specific exergy (MJ/kg CO_2_eq)	purity CH_4_ (%)	purity CO_2_ (%)	specific exergy (MJ/kg CO_2_eq)
AC (1)	96.1	1	2.3	93.3	1.1	8.4	82	1.8	96.6
Cd-MOF (1)	90.8	2.5	3.9	75.1	4.4	13.3	20.4	6.5	192
AC (3)	35.5	58.3	5.8	23.5	64.8	17.1	7.5	66.9	102.8
AC (2)	49.5	28.6	3.2	33.2	26.7	10.8	7.06	18.3	104.6
Cu-MOF (3)	2.6	67.9	42	0.9	54.6	132.9	0.07	31.2	96.6
SAPO (1)	2.7	45.4	23.3	1	65.9	25.8	0.03	38.3	68.7
Cu-MOF (5)	1.37	46.6	42.7	0.8	52	63.2	0.02	90.1	22.11
Zn-MOF (1)	0.5	39.1	19	0.1	39.5	25	0.007	96.1	12.5
Cu-MOF (4)	1.7	50.2	26.4	0.4	26.9	95.7	0.001	13	119.1
Cu-MOF (6)	3.7	71.1	6.6	2	59	13.1	0.5	66.5	23.4
Ca-MOF (1)	51	26.5	3.1	49	33.4	9.8			
Cu-MOF (2)	3.5	67.8	7.6	1.1	78.6	10.1			
SAPO (2)	1.1	40.7	27	0.4	60.5	3.5			
Cu-MOF (1)	81.7	1.3	9.5						

a Results Are Reported for the Case of Maximum Purity of CH_4_. Empty cells in the open barn and air removal cases indicate that the sorbent cannot deliver any separation, i.e., the cycle working capacity is negative. AC refers to Activated Carbon.

The ternary diagrams allow us to put the results into perspective of possible uses of the separation product. To this end, as shown in Figure 8a), we identify four main areas: (i) high CO_2_ purity (higher than 95 vol %), which is, in first approximation, suitable for direct underground storage; (ii) high CH_4_ purity (higher than 95 vol %), which is suitable for natural gas substitution, e.g., with direct grid injection; and (iii) CH_4_ concentration above the flammability limit (roughly 5% in air), which is suitable for combustion (we note that CH_4_ oxidation has a positive climate effect, but with a non-negligible termination effect^60^). The limits of the four areas are intended as indicative, since they may shift under different boundary conditions; and (iv) CH_4_ concentration below the flammability limit, which requires catalytic conversion.

Figure 8b–d show the results of the material screening for the three different feed mixtures. For each material, two points are shown in the ternary graphs, which are representative of the maximum purity and minimum specific exergy along the Pareto front obtained from optimization. Each material type is depicted with a unique shape and colored according to the exergy consumption. The performance of the maximum CH_4_ purity point with the corresponding exergy consumption is also summarized in Table 4. To provide a reference for the magnitude of the reported exergy values, it is worth noting that the *exergy* demand of DAC processes typically lies in the range of 1.5–3.7 MJ kg_CO_2__^–1^, corresponding to a heat consumption of 6–15 MJ kg_CO_2__^–1^.^15^

Several materials are identified as possible CH_4_ removal of sorbents, however, the number of viable materials decreases with decreasing concentration (13 for 100 ppm of CH_4_, 12 for 25 ppm of CH_4_, and 9 for 2 ppm of CH_4_). Interestingly, some sorbents stand out as suitable across various inlet CH_4_–CO_2_ concentration levels: activated carbon, Cd- and Cu-based MOFs, and SAPO zeolites. When comparing the different feed mixtures, it becomes apparent that the exergy consumption rises sharply at lower concentrations (note that the color map ranges differ among panels (a), (b), and (c)). This is consistent with the logarithmic nature of the minimum thermodynamic work of separation and makes the separation from air extremely challenging. Moreover, the two Pareto points per material are generally similar in exergy consumption, while results can differ significantly in terms of purity; in all cases, the exergy consumption increases as the CH_4_ concentrations in the product increase. Table S5 shows the optimal operating conditions associated with the points shown in Table 4. It is possible to note that (i) the adsorption temperature is in most cases close to the lower bound, revealing that the process benefits of operating at low *T* (we note that a lower temperature might be further beneficial in terms of exergy per mass of product, but challenging to implement in distributed, compact applications); (ii) the desorption temperature is often below the upper bound, revealing that exergy can be minimized by limiting heating while benefiting of heat integrated cooling (this is confirmed by the minimum exergy points, where exergy is minimized by decreasing the desorption temperature); and (iii) the vacuum pressure is in most cases close, or very close, to the lower bound.

More detailed insights can be drawn from examining each panel.

100 ppm of CH_4_-1200 ppm of CO_2_ (Figure 8b). Activated carbons and MOFs, e.g., Cd-MOF(1), perform the best in terms of CH_4_ product purity (>5%) and exergy consumption (2.3–6.0 MJ/kg CO_2_eq). Moreover, the high purity of methane in these cases makes combustion of the product a viable option that can be easily implemented within the process while also enabling energy recovery: considering a simple energy balance that includes the Carnot efficiency as second-law conversion factor, combusting the product gas can supply approximately 15–20% of the total exergy demand of the process. On the other hand, from a climate perspective, the CO_2_–CH_4_ costorage would be a preferred application; in this case, SAPO(1) and Zn-MOF(1) offer better opportunities for higher CO_2_ purity (>95%). A remark on the cases where the purity of CH_4_ is higher than that of CO_2_ is needed, especially given the higher concentration in the feed of the latter compared to the former. For equilibrium models, the purity in the product depends on the cyclic adsorption capacity of each component: the component with the highest cyclic capacity will have the largest mass in the product and therefore the highest purity. Accordingly, the four sorbents with CH_4_ purities higher than those of CO_2_ in the product feature higher cyclic capacities for methane than for CO_2_. This happens because of a combination of two reasons: (i) different from CO_2_, the sorbent retains almost no methane at the desorption conditions (see Figure S8 and explanation in the SI), and (ii) the sorbent adsorbs similar or more methane during adsorption (see Figure S9 and explanation in the SI). While (i) can indeed be possible, we do not see physical reasons for (ii) to happen for these four sorbents: CO_2_ should adsorb more in the Henry region, even when at subambient conditions (we note that this is not the case for CH_4_–N_2_, whose selectivity can invert at subambient temperature as shown by Eyer et al.^24^). Instead, the reason for the higher adsorption can be found in the isotherm fitting at low partial pressure, where experimental data are not available: without data in the region, the fitting can be overall very good but with CH_4_ adsorption overestimated compared to CO_2_. Therefore, while the four sorbents remain promising for their remarkable capacity of removing CH_4_ over N_2_, the methane-to-CO_2_ ratio in the product needs to be reassessed with isotherms that include low-pressure data. Similar reasoning also applies to the following cases with a lower CH_4_ concentration.

25 ppm of CH_4_, 700 ppm of CO_2_ (Figure 8c). Overall, the same material candidates are identified as in the higher concentration case; however, fewer materials can achieve a significant CH_4_ purity in the product, while the exergy consumption increases to 8.4–17.1 MJ/kg CO_2_eq. Notably, high CO_2_ purity is still theoretically possible, leaving essentially all product uses open depending on the sorbent, as in the higher concentration.

2 ppm of CH_4_-400 ppm of CO_2_ (Figure 8d). Results are significantly different when capturing CH_4_ and CO_2_ directly from air. Producing methane at high purity requires a large exergy expenditure (more than 96.6 MJ/kg of CO_2_eq), which makes the process hardly viable. On the other hand, materials more selective toward CO_2_, e.g., Zn-MOF(1), allow us to capture CO_2_ with a reasonable exergy demand of 12.56 MJ/kg CO_2_eq while also having a trace amount of CH_4_ in the product.

When comparing the screening results, it is possible to see some trends with respect to the class of different sorbents: carbon-based materials perform well in producing higher purity methane, whereas zeolites and SAPOs perform better in achieving higher purity CO_2_. This is rooted in the properties of the material. Carbon materials have a high methane adsorption capacity combined with moderate CO_2_/CH_4_ selectivity, making them effective for methane capture. In contrast, zeolites and SAPOs offer high CO_2_/CH_4_ selectivity and high CO_2_ adsorption capacity, resulting in the production of high-purity CO_2_. Eventually, determining the optimal sorbent and application requires a comprehensive analysis of the entire energy system, encompassing both capture and downstream utilization.

## Discussion

In this work, we investigated methane removal from dilute sources by employing a model. Equilibrium models have proven beneficial in the early-stage development of adsorption processes, especially for deriving intuition and understanding of the process, for preliminary screening of possible materials, and for identifying the limits of achievable performance. This matches well the scope of this work, which for the first time assesses methane removal from very dilute sources for climate mitigation. Moreover, the equilibrium model developed in this work was shown to align well with more detailed 1D models. Nevertheless, and as in all modeling work, several limitations apply. In the following, we discuss the main limitations, compare to other studies, and provide some directions for future research.

### Limitation: Isotherms Data and Modeling

It is important to note that the present study uses isotherm data available in the open scientific literature. This has three relevant consequences. (i) Many of the experimental setups used to report isotherms were not thought for very dilute CH_4_ concentrations. While thermodynamic-consistent isotherm models allow extrapolation of points at low concentration, having experimental values in this region is required to improve accuracy, especially for 0D models, which rely on prediction of the working capacity. This is particularly important for the CO_2_–CH_4_ selectivity in the Henry region: under similar adsorption–desorption kinetics, the product composition will be strongly affected by the isotherm shape at low partial pressure. (ii) The isotherm data are not always available for all components in the mixture. Therefore, for a few sorbents, simplifications were required to predict the behavior of CO_2_ or N_2_ (while methane isotherms were always available for the screened sorbents). (iii) Multicomponent mixture behavior and gas competition is neglected. In this study, we investigated CH_4_ removal using single-component gas isotherms. This simplification was necessary due to the lack of consistent isotherm data throughout the database for the three species on the same sorbent, and it is in line with the scope of the 0D model, i.e., to develop a preliminary process understanding and to identify promising sorbents. However, and especially in the case of weak gas-surface interactions, N_2_, CH_4_, and CO_2_ will compete. In order to obtain more insights into the difference between unary isotherms and multicomponent behavior, we have compared the gas uptake for the Toth single isotherm equation, which is often adopted by the model to fit the NIST data, and the IAST theory. The IAST adsorption was calculated using PyIAST, an open-access tool developed by Simon et al.^61^ When doing so, we adopted the analytical Langmuir model, with the exception of CO_2_ for Cu-MOF(6), where the Langmuir fitting is unsatisfactory. Figure 9 shows the comparison for two exemplary sorbents where data for all components were available (Cu-MOF(6)^55^ and Cu-MOF(7)^51^). Figure 9a,c show the Toth isotherm for the three components at 298 K, while the symbols show the IAST gas uptake for the feed conditions of the semiclosed barn case. For Cu-MOF(6), the IAST prediction is close to the pure gas isotherm for all components, while for Cu-MOF(7), a deviation is found for CO_2_ and CH_4_. This can be better evaluated from Figure 9b,d, where the gas uptake is compared at feed conditions for the two materials. It can be noted that the two models are indeed very close for Cu-MOF(6), with a growing deviation at a lower adsorbed capacity. Consequently, Cu-MOF(7), which has less favorable isotherms, features a significant difference in the CO_2_ and CH_4_ uptake (it is worth stressing that Cu-MOF(7) was not among the best sorbents). The comparison between unary isotherms and the IAST theory shown here provides reassurance for the overall material screening, i.e., the capability of identifying promising sorbents. This is further reinforced by the additional comparison shown in Figure S3, which reports the recovery-purity Pareto front for Cu-MOF(6) obtained with a 1D model^15^ using the single Toth isotherm and the extended Langmuir: the figure shows that, while the two isotherm models result in a 10–20% performance difference, the quantification with unary isotherms is sufficiently good to identify Cu-MOF(6) as a promising candidate in an early stage sorbent screening. However, the IAST-unary isotherm comparison also reveals that deviations can be significant, especially at low partial pressures. Accordingly, competition behaviors in the Henry region will affect the quantitative performance of the sorbents and their ranking.

**Figure 9:**
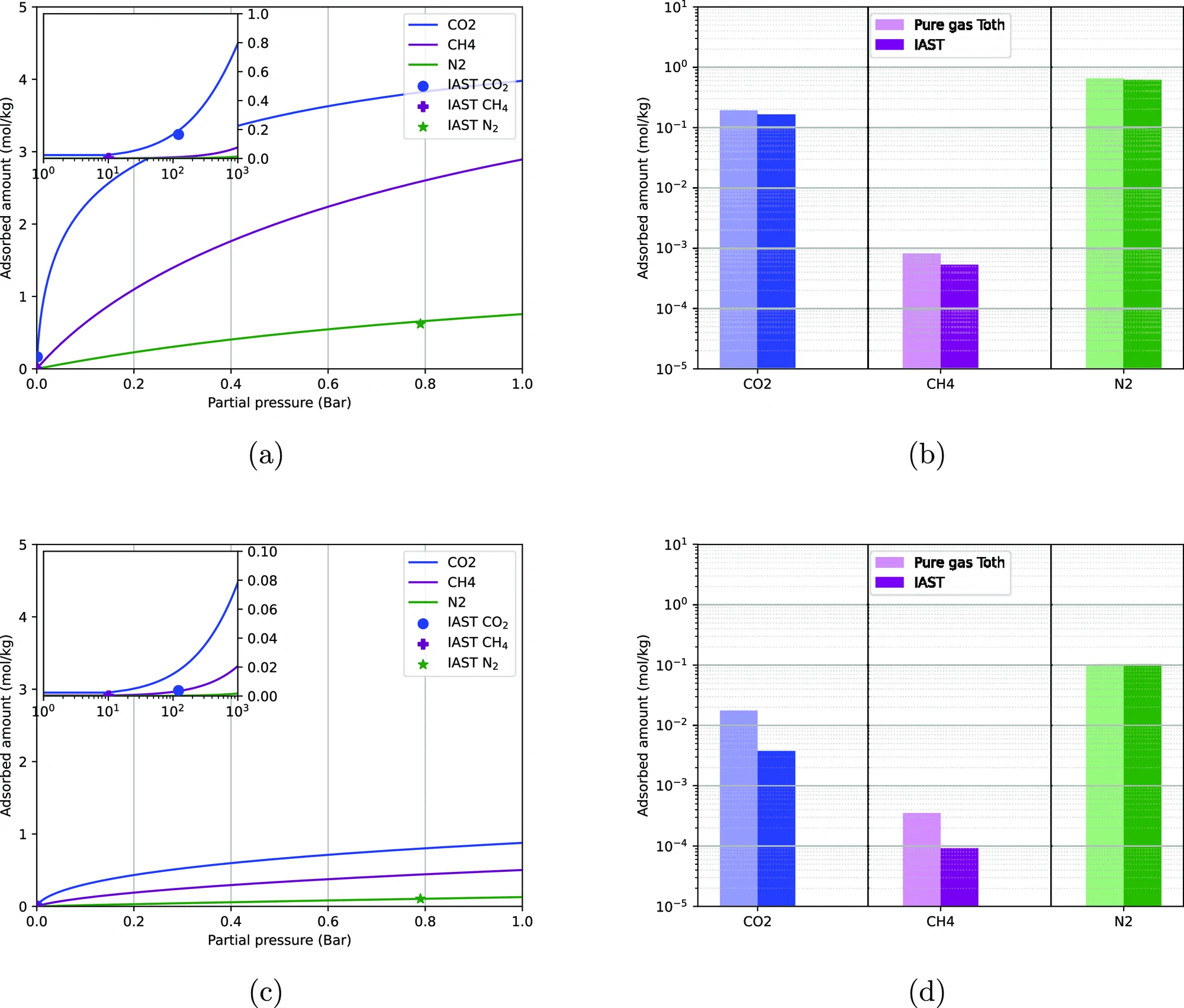
Comparison of pure gas and IAST gas adsorption for the semiclosed-stable composition at 298 K. Top (a), (b): results for Cu-MOF(6).^55^ Bottom (c), (d): results for Cu-MOF(7)^51^ (not included in Figure 8). Figures (a) and (c) show the Toth pure gas isotherms (lines) and the IAST gas uptake (symbols) at feed conditions and 298 K; the inset shows the low pressure region (note the log pressure axis in [Pa]). Figures (b) and (c) shows the comparison of the gas uptake for the Toth unary (left bar) and IAST (right bar) for the three components of the mixture at feed conditions. Figures S4–S7 show the comparison for the open barn and air.

### Limitation: The Role of Water and Oxygen

In this work, we also simplify how we treat oxygen and water. For the former, we assumed the same adsorption behavior for O_2_ and N_2_, due to lack of information for O_2_, both in terms of adsorption and content in the inlet gas. While the O_2_ content is not expected to change the separation performance significantly (in fact, our simplification results in more N_2_ adsorption), the presence of O_2_–CH_4_ mixture poses a hazardous threat that must be adequately handled in a possible design phase. From this perspective, it is however worth stressing that the autoignition temperature of CH_4_ in air is significantly higher compared to the regeneration temperature of the process (537 vs 120 °C).

When considering H_2_O, the process illustrated above allows for deep removal of H_2_O before the adsorption bed. Therefore, we do not consider water in the mixture entering the VPSA cycle, removing the need for water adsorption isotherms, one of the key challenges in modeling DAC processes. However, while combining low-temperature cooling and a guard bed can significantly decrease the amount of water, H_2_O content is likely to remain similar to that of CH_4_. At similar conditions (subambient *T*, low RH), H_2_O can still compete with CH_4_ and CO_2_ adsorption depending on the type of sorbent. Trace water might preferentially adsorb on hydrophilic sorbents (e.g., zeolites and open-metal-site MOFs), leading to significant site blocking and long-term deactivation, whereas hydrophobic materials such as activated carbons and CALF-20 might remain largely unaffected. Remarkably, several of the most performing CH_4_ sorbents identified, such as zeolites and Cu-based MOFs, can experience degradation in the presence of trace water content. This would have severe consequences on the process design, as water would directly affect the viability of the separation. This open point needs to be tackled with stability experimental tests. Overall, further understanding on the role of O_2_ and H_2_O can only be obtained with dedicated experimental campaigns.

### Limitation: Equilibrium Model

It is important to note that, in general, 0D models do not provide particularly precise predictions of the process performances. Separation by adsorption is controlled by transport phenomena (heat and mass), and equilibrium is a useful assumption that allows to develop initial knowledge and/or to tackle computational bottlenecks (both apply in this study). However, by removing the time dimension, equilibrium models do not allow to describe the limits set by transport phenomena: on the one hand, it is not possible to include mass transfer associated with material and contactor characteristics; on the other hand, it is not possible to include the engineering challenges arising from heat transfer, such as those associated with designing efficient heating/cooling systems, and those associated with matching heating and cooling times for continuous cyclic operation. Therefore, it is important not to draw final conclusions on the best/worst sorbent using the results presented here. Rather, 0D models help in identifying promising sorbents and in obtaining a ballpark number of their process performance beyond simple sorbent metrics, thereby facilitating the next validation and design steps: The results generated here serve as a starting point for new experimental testing and for more detailed 1D or 2D rate-based models. Moreover, it is important to stress that the performance of CH_4_ separation might change when changing the objective functions in the optimization, e.g., by maximizing recovery and purity, or by maximizing productivity and minimizing energy consumption. While in this work we opted for purity and exergy consumption, this choice should be re-evaluated when integrating this separation in a specific environment (farm) or with a downstream process (CH_4_ conversion).

### Comparison with CH_4_ Separation from More Concentrated Streams

While there are no works in the literature that have investigated methane removal from very dilute sources, some insights can be gained comparing this work with studies that address CH_4_ removal from % level streams, such as coal mine ventilation air. Bae et al.^19^ showed experimentally that methane purity could be increased from 3 to 4% in the feed (methane diluted in air) to 32–37% in the product using carbon composites and a double VSA+TVSA arrangement. Using process modeling and optimization, Guo et al.^20^ showed that CH_4_ could be enriched to 70% starting from a 5% CH_4_–N_2_ feed combining ionic liquid zeolites with dual reflux VSA. Yang et al.^22^ reached 90% purity from a 10% feed using carbon molecular sieves. In all cases, emphasis was put on CH_4_–N_2_ separation, with no investigation of the role of CO_2_. The enrichment factors that we compute in this work, which can be derived from Table 4, are generally higher; for example, for semiclosed barns we have values ranging from 50 (Zn-MOF(1)) to thousands (activated carbon(1)). Likely, these higher values are achieved because of higher energy consumption. For example, Guo et al.^20^ report an energy consumption of around 41 MJ/kg CH_4_ for an enrichment factor of 14, while in this study we find enrichment factors beyond 50 with energy consumptions in the range of 64–1200 MJ/kg CH_4_ (allocating all energy consumption to methane and converting the energy consumption in Table 4 from MJ/kg CO_2_ to MJ/kg CH_4_).

### Future Research

In light of these limits, future research should give priority to improving the experimental sorbent characterization and demonstrating the separation experimentally. From a modeling perspective, rate-based process modeling is a necessary step to improve the understanding of the separation and the role of mass transfer. With more precise isotherms data available, modeling should use competitive isotherms; the ideal adsorption theory and its simplification to the extended Langmuir isotherm could be reasonable approaches. This might result in different quantitative performance of the sorbents identified here, i.e., their position in the ternary diagram and specific energy consumption; the overall identification of promising sorbents is instead expected to remain robust. From a technical implementation perspective, further R&D should also include water, oxygen, and impurities found in typical input streams, e.g., NOx, VOCs, NH_3_, as they may strongly affect the type of sorbents that can be practically adopted. Moreover, the technical developments on the separation process should directly feed into climate and integrated assessment models so as to best identify the role of this solution in the broader context of climate change mitigation.

## Conclusions

In this study, we investigated the separation of CH_4_ from dilute emission sources, including ambient air, as a means of mitigating climate change. Given the novelty of this application, we developed an equilibrium-based 0D model that enables the rapid simulation of VTSA processes while retaining a physics-based description of the underlying phenomena. This approach provides insights into the fundamental process behavior and performance limits. The model was integrated with rigorous process optimization and applied to high-throughput sorbent screening using adsorption data from the ARPA-E/NIST database, allowing the identification of promising sorbents for methane capture from dilute streams. The 0D model includes equilibrium-based mass and energy balances for ternary CH_4_, CO_2_, and N_2_ mixtures with pure-component isotherms and was successfully verified against a detailed 1D rate-based model under equivalent input and boundary conditions. Moreover, in this work, we developed a first process concept for methane removal from dilute sources, in which (i) adsorption is performed, when beneficial, at subambient temperatures; (ii) heat integration is employed to reduce the cooling duty; and (iii) water is fully removed as byproduct of the subambient cooling.

Using the 0D model and this process concept, we investigated three representative CH_4_–CO_2_ concentration scenarios: 100 ppm of CH_4_—1200 ppm of CO_2_, characteristic of semiclosed dairy farms; 25 ppm of CH_4_—700 ppm of CO_2_, characteristic of open dairy barns, rice cultivation, and wetland emissions; 2 ppm of CH_4_—400 ppm of CO_2_, representative of direct air capture conditions. In all cases, different sorbents were identified as promising, with three different material classes highlighted from the screening: Activated Carbons, MOFs, and Zeolites. AC performed particularly well when delivering higher purity methane, while zeolites led to higher purity CO_2_. MOFs filled the remaining separation spectrum depending of the metal cluster. When looking at the three concentration cases, the key findings can be summarized as follows:

- 100 ppm of CH_4_—1200 ppm of CO_2_: Activated carbons, Cu-MOF, and Cd-MOF emerged as promising sorbents, achieving high CH_4_ purity at an exergy demand of ~4.0 MJ/kgCO_2_eq. The methane-rich product could be combusted directly in air, potentially recovering up to 20% of the capture process’s exergy requirement. For applications favoring high-purity CO_2_ or CO_2_–CH_4_ cocapture, sorbents such as SAPO, zeolites, and MOFs were more suitable, yielding streams containing a few vol % CH_4_.
- 25 ppm of CH_4_—700 ppm of CO_2_: Activated carbons and Cd-MOF again performed best, though with a reduced average CH_4_ purity and a higher exergy demand of ~12 MJ/kgCO_2_eq
- 2 ppm of CH_4_—400 ppm of CO_2_: Under highly dilute conditions, only a few sorbents, where identified as promising candidates, all with a substantially higher exergy demand of 120 MJ/kgCO_2_eq, reflecting the thermodynamic challenges of direct CH_4_ capture from air.

It should be noted that low-pressure adsorption data for both CH_4_ and CO_2_ remain scarce in the literature: the limited experimental characterization in the Henry regime directly influences the calculated CH_4_–CO_2_ distribution in the product. This is particularly important for cases with higher CH_4_ purity in the product: in the absence of experimental points at low partial pressure, isotherm fitting can result in unrealistic CH_4_ selectivity. Moreover, while the comparison with the IAST competitive isotherm model shows reassurance for the overall sorbent screening, competitive adsorption effects in the Henry regime not accounted for with pure-component isotherms will alter the material ranking.

Overall, our modeling results indicate that methane can potentially be captured from dilute emission sources using existing and in some cases commercially available sorbents. Methane capture is a particularly compelling short-term mitigation strategy: methane is both a potent short-lived climate pollutant and an energy carrier. This dual role enables the design of partially energy-autonomous capture systems that could contribute to near-term climate mitigation and help avoid temperature peaks. It is however, important to note that, given the different behavior of CH_4_ compared to CO_2_ (role on short- vs long-term climate, GHG lifetime), the role of methane removal technologies and their deployment must be assessed with models that go beyond the technical performance presented here, including climate feedback loops and alternative reduction pathways.

Nevertheless, further research is required before firm conclusions can be drawn. Key needs include experimental characterization (e.g., adsorption isotherms, mass transfer), demonstration, and detailed process modeling (1D with competitive isotherms and CFD simulations). Additionally, opportunities for synergistic coadsorption of CO_2_ and CH_4_ in DAC-like processes merit more thorough investigation.

## Supplementary Material



## Notes

Additional information on assumptions, methods, and results is available online in the Supporting Information file. Data and codes are available at https://github.com/UU-ER/SorbentScreening_0Dmodel_VTSA (PDF)
