# Supplementary material for: Adsorption-Based Methane Removal from Dilute Non-fossil Sources: Process Concept and Sorbent Screening
Source: Ind Eng Chem Res. 2026 Aug 5;65(32):17298–314. doi: 10.1021/acs.iecr.5c05045 (PMC13502053; doi:10.1021/acs.iecr.5c05045)
Supplement: Supplementary file 1 [file ie5c05045_si_001.pdf]

Supplementary Information

Adsorption-Based Methane Removal from Dilute Non-Fossil Sources: Process Concept and Sorbent Screening

Kian Karimi[a], Matteo Gazzani[a,b,⋆]

[a] Copernicus Institute of Sustainable Development, Utrecht University, Princetonlaan 8a, 3584 CB, Utrecht, The Netherlands

[b] Inorganic Membranes and Membrane Reactors, Sustainable Process Engineering, Chemical Engineering and Chemistry, Eindhoven University of Technology, 5612 AP Eindhoven, The Netherlands

⋆Corresponding author. Email: m.gazzani@uu.nl

1 Isotherm Model

The gas isotherm models used in our simulations to describe the adsorption equilibrium are provided in the table S1.

Table S1: Table with isotherm models and their equations (continued on next page)

Model	Equation
Toth	$$q_i = n_s \frac{bpy_i}{\left(1 + (bpy_i)^t\right)^{1/t}} \quad \text{(S1)}$$ $$n_s = n_{s0} \exp\left(X_i\left(1 - \frac{T}{T_0}\right)\right) \quad \text{(S2)}$$ $$b = b_0 \exp\left(\frac{\Delta H}{RT_0}\left(\frac{T_0}{T} - 1\right)\right) \quad \text{(S3)}$$ $$t = t_0 + \alpha\left(1 - \frac{T_0}{T}\right) \quad \text{(S4)}$$
Langmuir -Freundlich	$$q_i = n_s \frac{(bpy_i)^{1/t}}{\left(1 + (bpy_i)^{1/t}\right)} \quad \text{(S5)}$$ $$n_s = n_{s0} \exp\left(X_i\left(1 - \frac{T}{T_0}\right)\right) \quad \text{(S6)}$$ $$b = b_0 \exp\left(\frac{\Delta H}{RT_0}\left(\frac{T_0}{T} - 1\right)\right) \quad \text{(S7)}$$ $$t = t_0 + \alpha\left(1 - \frac{T_0}{T}\right) \quad \text{(S8)}$$

S-shaped	$$q_i = q_L(1-w) + q_U w \quad \text{(S9)}$$ $$q_L = \frac{q_{L,0} b_L(py_i)}{1+b_L(py_i)} \quad \text{(S10)}$$ $$b_{L/U/H} = b_{L/U/H,0} \exp\left(\frac{\Delta H_{L/U/H}}{RT}\right) \quad \text{(S11)}$$ $$w = \frac{\exp\left(\frac{\log(py_i)-\log(p_{\text{step}})}{\sigma}\right)}{\left(1+\exp\left(\frac{\log(py_i)-\log(p_{\text{step}})}{\sigma}\right)\right)^{\gamma}} \quad \text{(S12)}$$ $$p_{\text{step}} = p_{\text{step},0} \exp\left(-\frac{\Delta H_{\text{step}}}{R}(\frac{1}{T0}-\frac{1}{T})\right) \quad \text{(S13)}$$ $$\sigma = X_{i1} \exp\left(X_{i2}\left(\frac{1}{T0}-\frac{1}{T}\right)\right) \quad \text{(S14)}$$ $$q_U = \frac{q^*_{U,0} b_U(py_i)}{1+b_U(py_i)} + b_H(py_i) \quad \text{(S15)}$$

2 0D Model

For the 0D model, the mass and energy balances per step of the VTSA process are summarized in the table S2. It is important to mention that the Blow-down and Adsorption steps are assumed to be isothermal.

2.1 0D model, Cooling cycle energy balance

Heat integration in the drying and cooling section of the process (refer to Figure 2 in the main article) is calculated according to the following energy balance, where we have reasonably assumed that the flow of air entering/leaving the separation cycle and its specific heat capacity are the same:

$$Q = \dot{N}C_p(T_3 - T_{ads}) = \dot{N}C_p(T_{amb} - T_1) \tag{S23}$$

Therefore,

$$T_3 - T_{ads} = T_{amb} - T_1 \tag{S24}$$

moreover, for the pre-cooling heat exchanger we have considered $T_{\text{amb}} - T_3 = 10\,^{\circ}\text{C}$, and for the cooling cycle a $\Delta T_{\text{min}} = 5\,^{\circ}\text{C}$.

Table S2: Summary of 0D equations

Step	Material/ Energy Balance	Equation
Blow-down	Material balance	$$N_{i,\text{total}}^{k-1} - y_i^k N_{\text{out}}^k = N_{i,\text{total}}^k \quad \text{(S16)}$$
	Energy balance	isothermal
Heating and vacuum	Material balance	$$N_{i,\text{total}}^{k-1} - y_i^k N_{out}^k = N_{i,\text{total}}^k \quad \text{(S17)}$$
	Energy balance	$$Q_{\text{heating}}^k = \sum_i (N_{i,s}^k - N_{i,s}^{k-1})\lvert\Delta H_{\text{ads,i}}^k\rvert + m_s c_{p,s}(T_{\text{des}}^k - T_{\text{des}}^{k-1}) \quad \text{(S18)}$$
Cooling and pressurization	Material balance	$$\sum_i N_{i,\text{total}}^{k-1} - y_{i,\text{feed}} N_{in}^{\text{Air},k} = \sum_i N_{i,\text{total}}^k \quad \text{(S19)}$$
	Energy balance	$$Q_{\text{cooling}}^k = \sum_i (N_{i,s}^{k-1} - N_{i,s}^k)\lvert\Delta H_{\text{ads,i}}^k\rvert + m_s c_{p,s}(T_{\text{des}}^k - T_{\text{des}}^{k-1}) \quad \text{(S20)}$$
Adsorption	Material balance	$$N_{i,\text{total}}(t_f) - N_{i,\text{total}}(t_0) = y_i^{\text{feed}} \int_{t_0}^{t_f} \dot{N}_{\text{feed}} dt - y_i(t_0) \int_{t_0}^{t_f} \dot{N}_{\text{waste}} dt = N_i^{\text{in}} - N_i^{\text{out}} \quad \text{(S21)}$$ $$N_{i,\text{total}}(t_f) = m_s q_i^* + y_i^{\text{feed}} \frac{p_{\text{amb}} V \varepsilon}{R T_{\text{amb}}} \quad \text{(S22)}$$
	Energy balance	isothermal

3 1D Model

The 1D model equations are provided in the table S3 while table S4 reports the bed specifications used for 1D simulations.

Table S3: Equations for 1D adsorption model

Description	Equation	
**Total mass balance**	$\epsilon_t \frac{\partial (p/T)}{\partial t} + \frac{\partial (up/T)}{\partial x} + \rho_b R \sum_{j=1}^{N} \frac{\partial n_j}{\partial t} = 0$	(S25)
**Component mass balance**	$\epsilon \frac{\partial (y_i p/T)}{\partial t} + \frac{\partial (u y_i p/T)}{\partial x} + \rho_b R \frac{\partial n_i}{\partial t} = 0, \quad i = 1, \ldots, N$	(S26)
**Mass transfer (Linear driving force model)**	$\frac{\partial n_i}{\partial t} = k_i \left(n_i^{\text{eq}} - n_i\right), \quad i = 1, \ldots, N$	(S27)
**Energy balance for the fixed bed**	$\left(\epsilon_t C_g + \rho_b C_s + \rho_b C_{\text{ads}}\right) \frac{\partial p}{\partial t} - \epsilon \frac{\partial p}{\partial t} + u C_g \frac{\partial T}{\partial x} - \rho_b \sum_{j=1}^{N} (-\Delta H_j) \frac{\partial n_j}{\partial t} + \frac{4 h_{\text{wall-bed}}}{d_i} (T - T_w) = 0$	(S28)
**Energy balance for the bed wall**	$\frac{\partial T_w}{\partial t} = \frac{4}{C_{p,w} \left(d_0^2 - d_i^2\right)} \left(d_i h_{\text{wall-bed}} (T - T_w) + d_0 h_{\text{fluid-wall}} (T_{\text{fluid}} - T_w)\right)$	(S29)
**Momentum balance (Ergun equation)**	$\frac{\partial p}{\partial x} = -\frac{150 \mu (1 - \epsilon_b)^2}{\epsilon_b^3 d_p^2} u - \frac{1.75 (1 - \epsilon_b)}{\epsilon_b^3 d_p} \rho \lvert u \rvert u$	(S30)

Table S4: Parameters for modeling the adsorption unit using the 1D model.

Parameter	Value
Length	0.045 m
Internal radius	0.005 m
External radius	0.0051 m
Material density	1590 kg/m^3
Bed density	375.4 kg/m^3
Heat capacity wall	2.457×10^6 J/(K m^3)
Heat transfer fluid/wall	20 W/(m^{2}K)
Heat transfer wall/ambient	100 W/(m^{2}K)

4 Verification

Figure S1 compares the 1D and 0D results in terms of molar fractions of CH_4 and CO_2 plotted versus temperature and pressure for ZIF69.

Figure S2 demonstrates the parity plots of the CO_2 purity, recovery and working capacity. The results show for most of the cases, the 0D and 1D predictions are in good agreement and stay in 10% range.

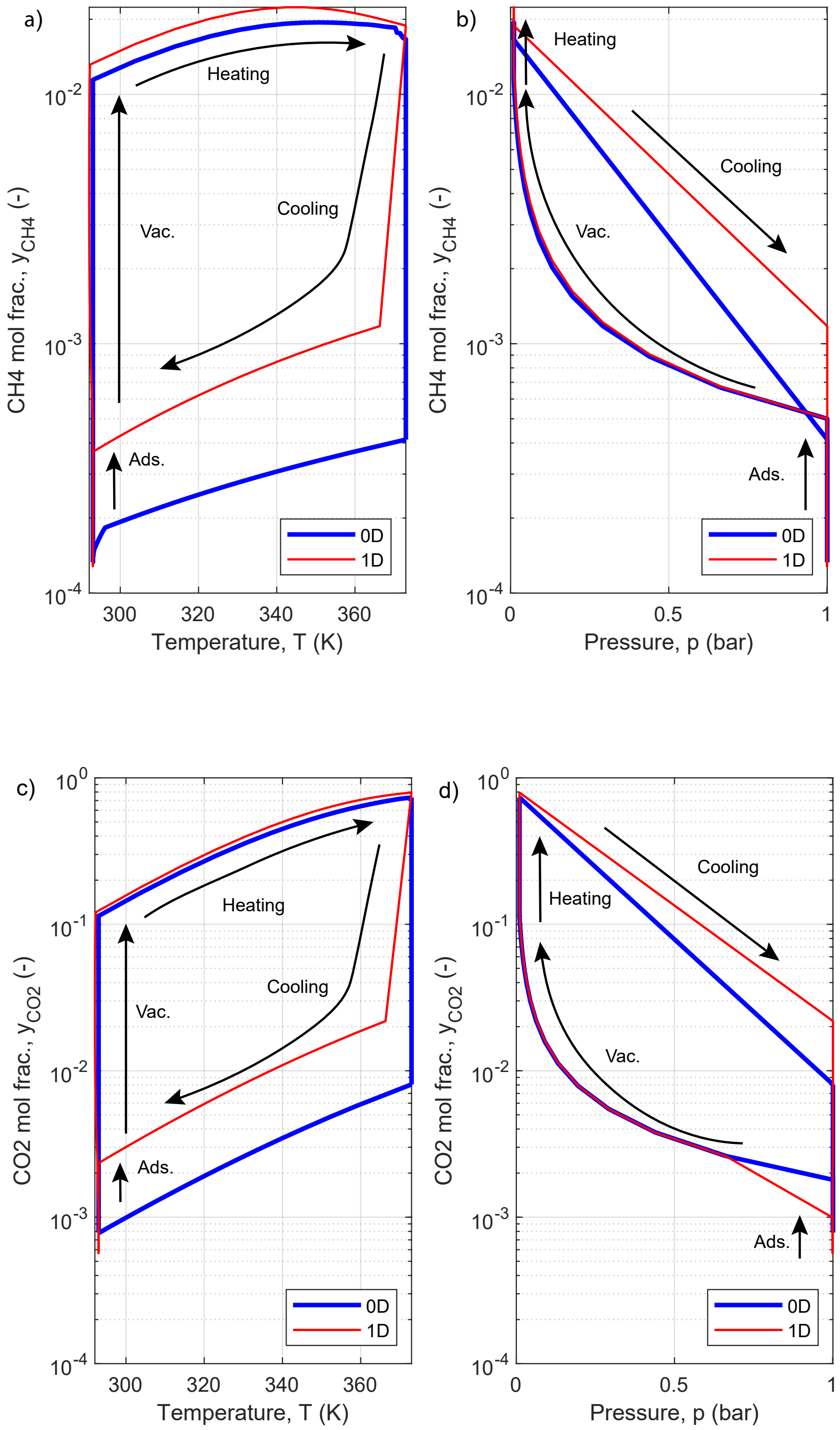

Figure S1: Mol frac. profiles of CH_4 (a,b) and CO_2 (c,d) over the temperature and pressure for the case of ZIF69. For the 1D model, the temperature, pressure and mol frac. plots refer to the values at the bed outlet considering cyclic steady sate conditions.

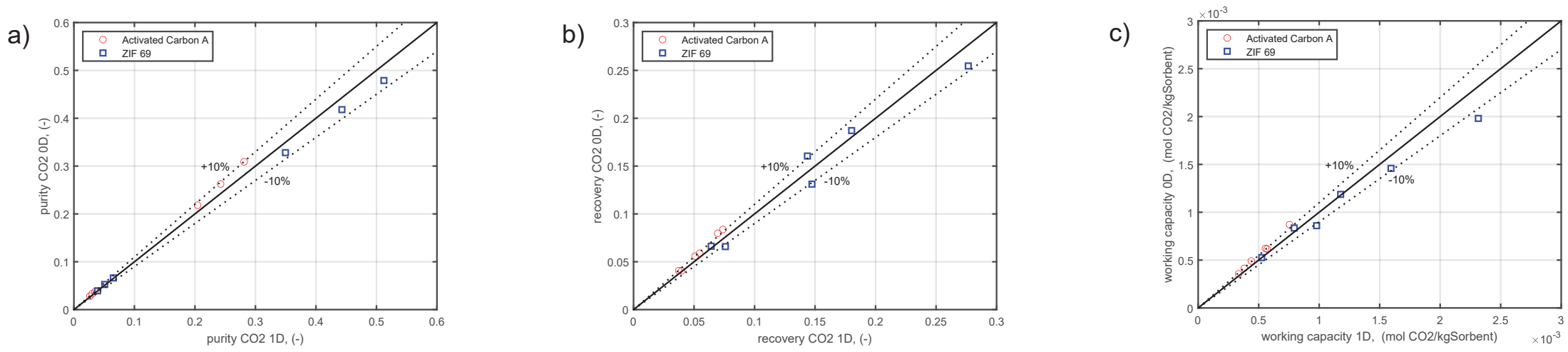

Figure S2: Parity plots of the performance parameters resulting from the 0D model and the 1D model. Each material is shown by a specific symbol and color, and each point features different input parameters for Tdes and pvac. The dashed lines show a margin of 10%.

5 Additional Results

Table S5 shows the optimal operating conditions for the maximum purity results reported in Table 6.

Figure S3 shows the different separation performance for Cu-MOF(6) when considering a unary gas isotherm (Toth model) and an extended Langmuir gas isotherm for the three components. As shown in the Figure, the difference between the two approaches noticeable but not relevant in the context of the scope of large scale screening of promising sorbents, i.e. the performance remain of interest when using an extended isotherm approach.

Figure S8 and S9 show the working capacity for CH_4 and CO_2 for two different activated carbons as function of the desorption temperature. In both cases, the higher working capacity of methane than CO_2 results in higher methane concentration in the product.

From Figure S8, it can be noted that the working capacity of CO_2 is substantially higher than CH_4at regeneration temperature below 330 K whereas the adsorption capacity of CH_4 becomes slightly larger at higher temperatures. This behavior is a result of the higher displacement of the CH_4 isotherm with temperature: it becomes relatively easier to clean the sorbent from CH_4 at higher temperatures than from CO_2.

On the other hand, Figure S9 shows that the adsorption capacity of methane is consistently higher than that of CO_2; this results from higher methane uptake in the Henry region due to limited availability of low pressure data. Physical intuition suggests CO_2 working capacity should be larger at lower desorption temperature.

Table S5: Optimal operating conditions for the different feedstock conditions

Material Name	100 ppm CH_4-1200 ppm CO_2			25 ppm CH_4-700 ppm CO_2			2 ppmCH_4-400 ppm CO_2		
	T_{Ads} (K)	T_{Des} (K)	p_{vac} (bar)	T_{Ads} (K)	T_{Des} (K)	p_{vac} (bar)	T_{Ads} (K)	T_{Des} (K)	p_{vac} (bar)
Activated Carbon (1)	253	344.2	0.01	253.3	354.6	0.01	253.4	372.6	0.01
Cd-MOF (1)	253.5	371.7	0.01	253	360	0.01	253.2	373	0.01
Activated Carbon (3)	253.4	370.2	0.01	253.2	372.4	0.01	253.1	373	0.01
Activated Carbon (2)	253.2	370	0.01	253.2	371.9	0.01	253	371.4	0.01
Cu-MOF (3)	293	338	0.01	293	348	0.01	257	362.6	0.05
SAPO (1)	254.6	320.4	0.03	255.5	325	0.02	253.1	371.7	0.06
Cu-MOF (5)	266.4	343.6	0.03	253.9	311	0.01	254.9	356.6	0.01
Zn-MOF (1)	255.4	331.6	0.1	253.2	342.5	0.1	254.4	364.3	0.01
Cu-MOF (4)	253	306	0.02	257.7	315.5	0.02	253.8	373	0.2
Cu-MOF (6)	253	345.3	0.01	253.1	329	0.01	253	364.9	0.01
Ca-MOF (1)	253.4	313.5	0.02	353.4	303	0.01	-	-	-
Cu-MOF (2)	253.4	316.4	0.01	253.2	335.6	0.01	-	-	-
SAPO (2)	293	308	0.01	266.3	311.3	0.15	-	-	-
Cu-MOF (1)	293	373	0.01	-	-	-	-	-	-

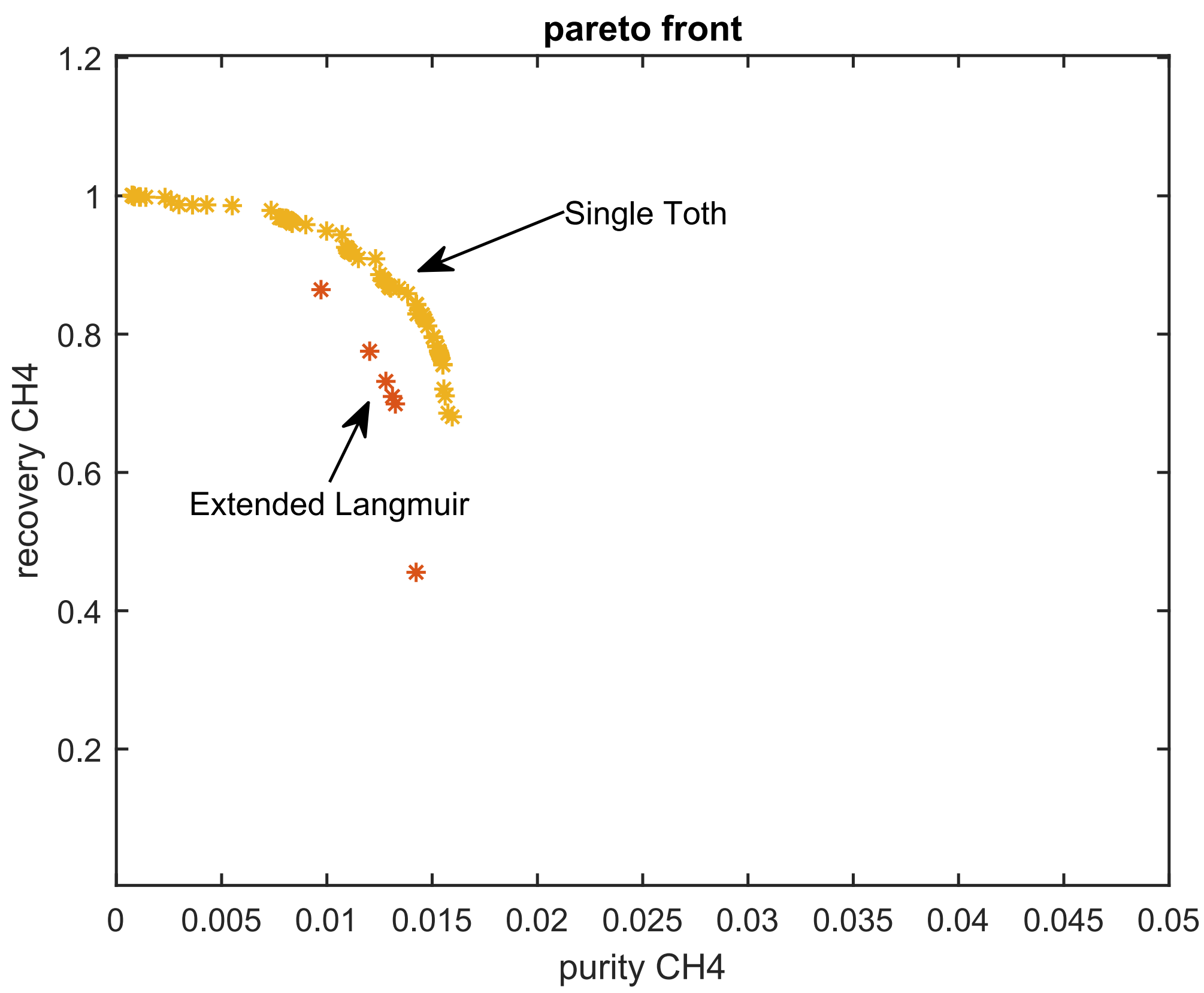

Figure S3: Purity-Recovery pareto for Cu-MOF(6) considering (i) unary gas isotherm with Toth model, and (ii) extended Langmuir gas isotherm.

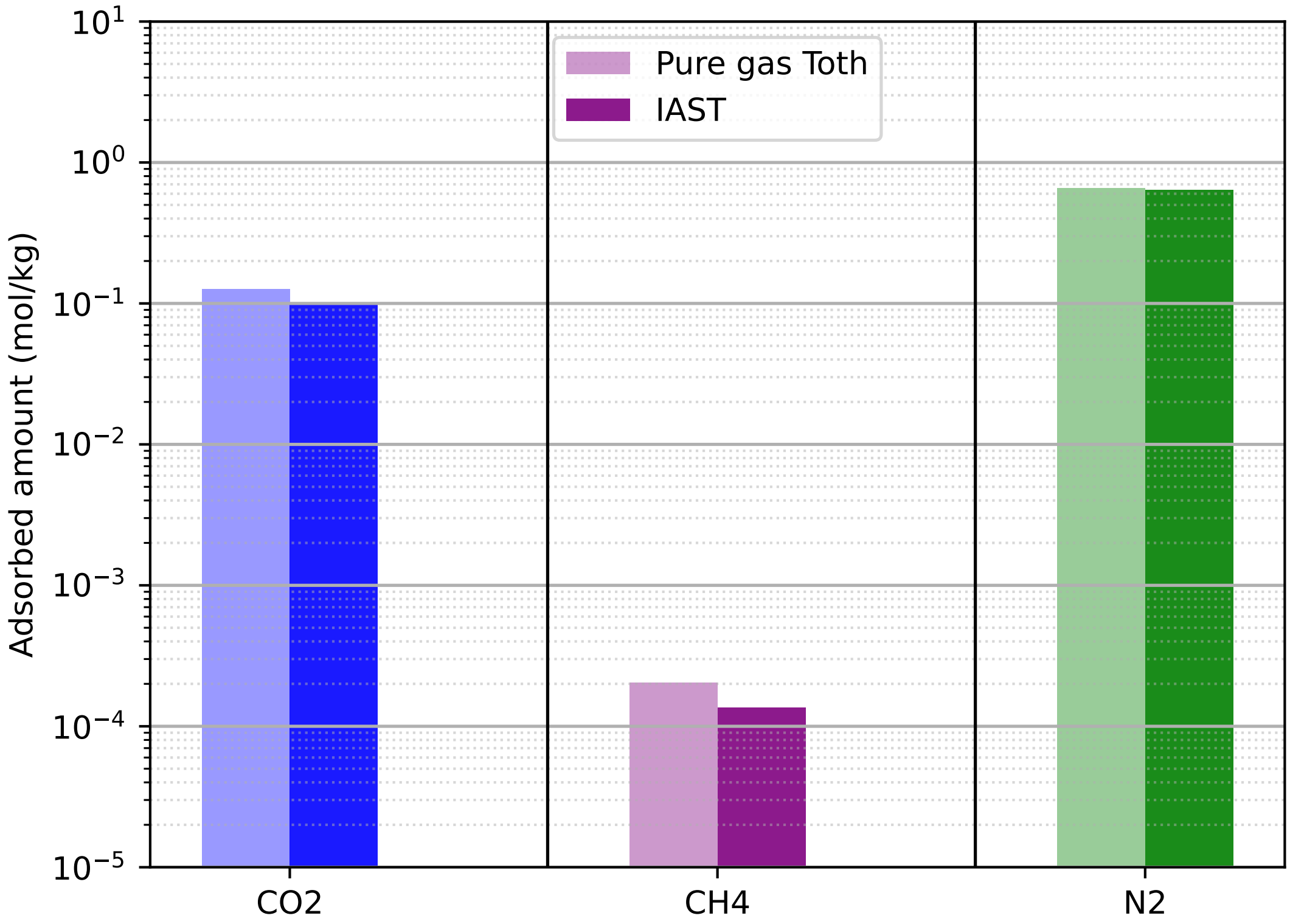

Figure S4: Comparison of pure gas and IAST gas adsorption for the open barn composition, Cu-MOF(6), 298 K.

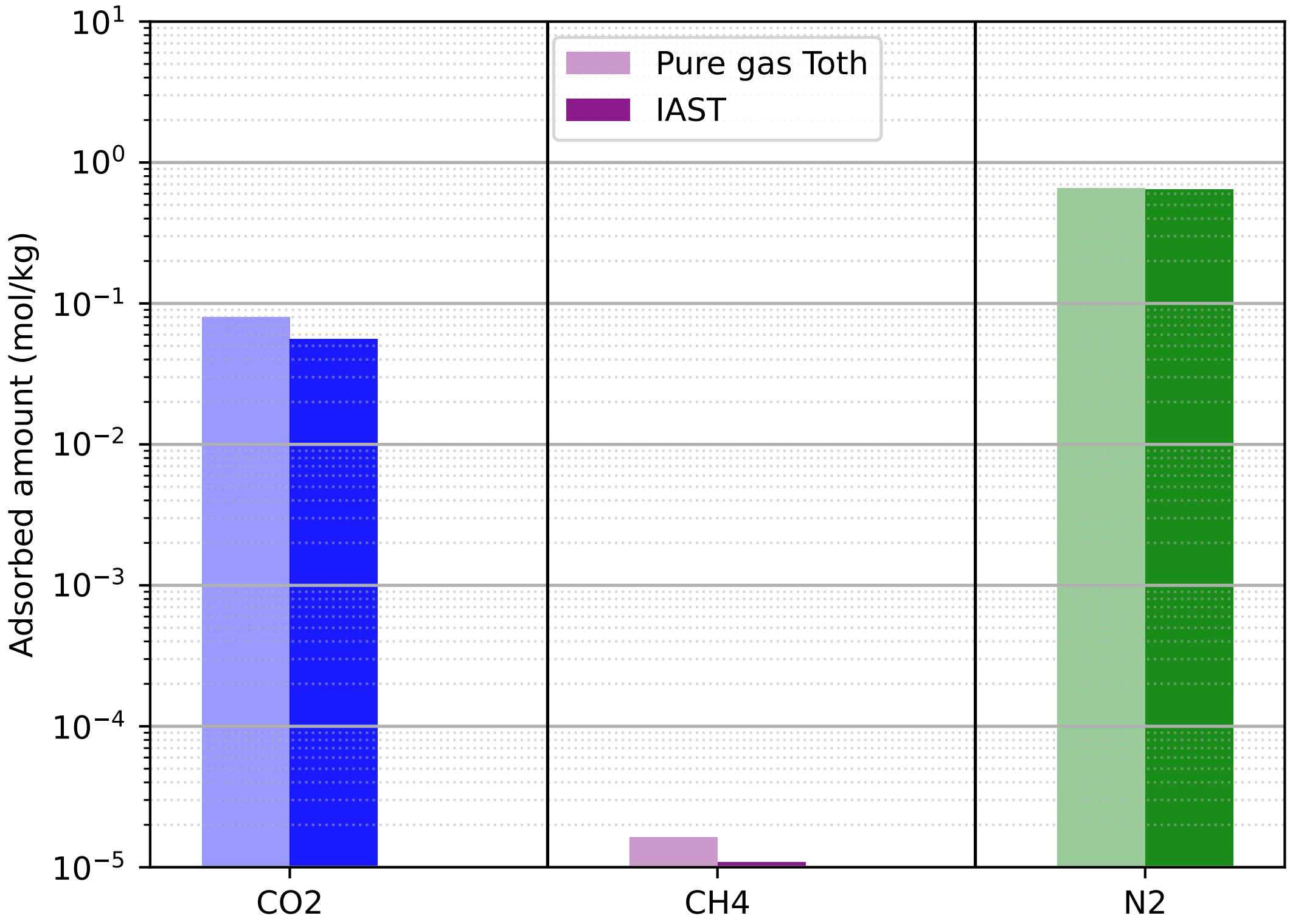

Figure S5: Comparison of pure gas and IAST gas adsorption for air, Cu-MOF(6), 298 K.

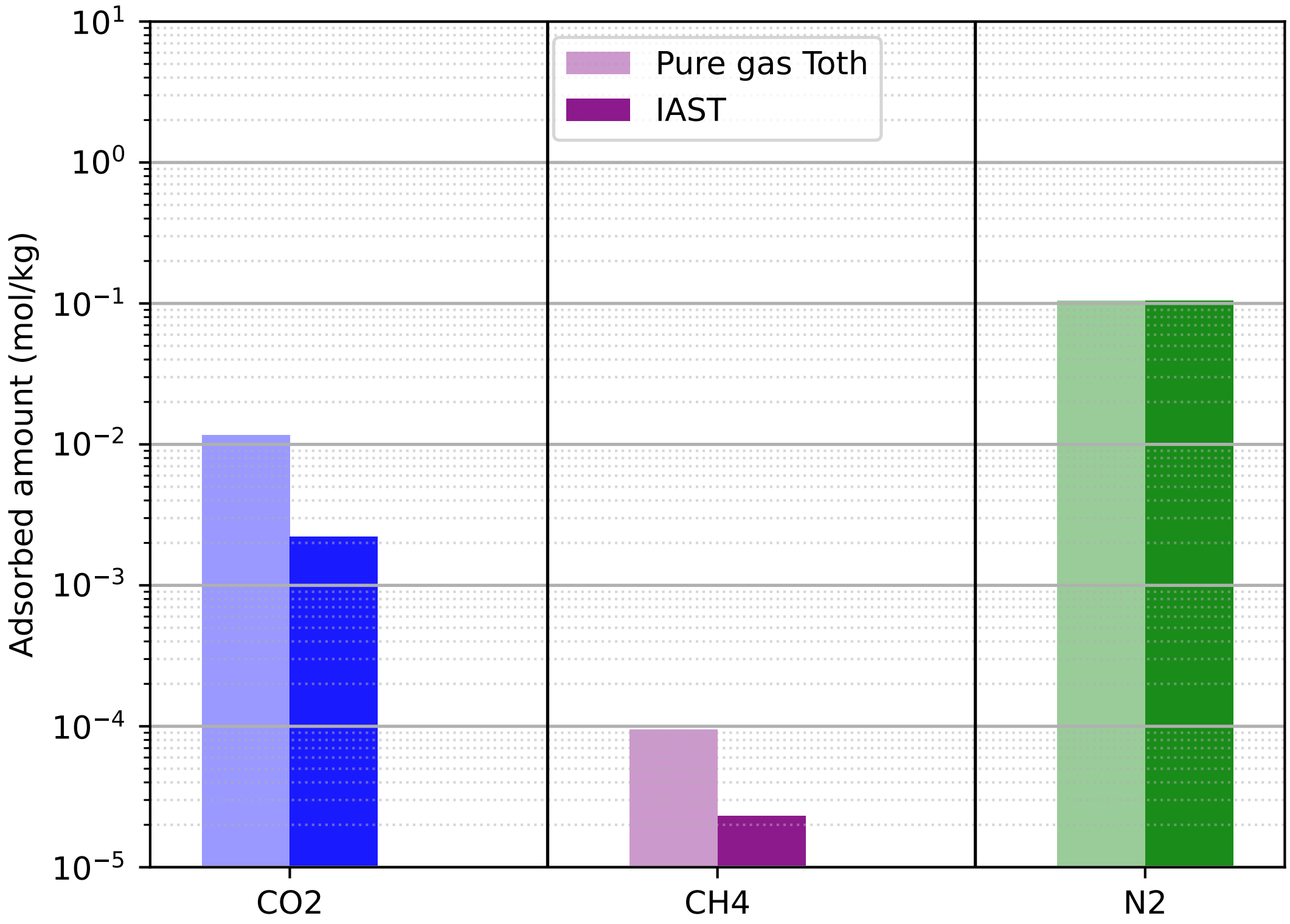

Figure S6: Comparison of pure gas and IAST gas adsorption for open barn composition, Cu-MOF(7), 298 K.

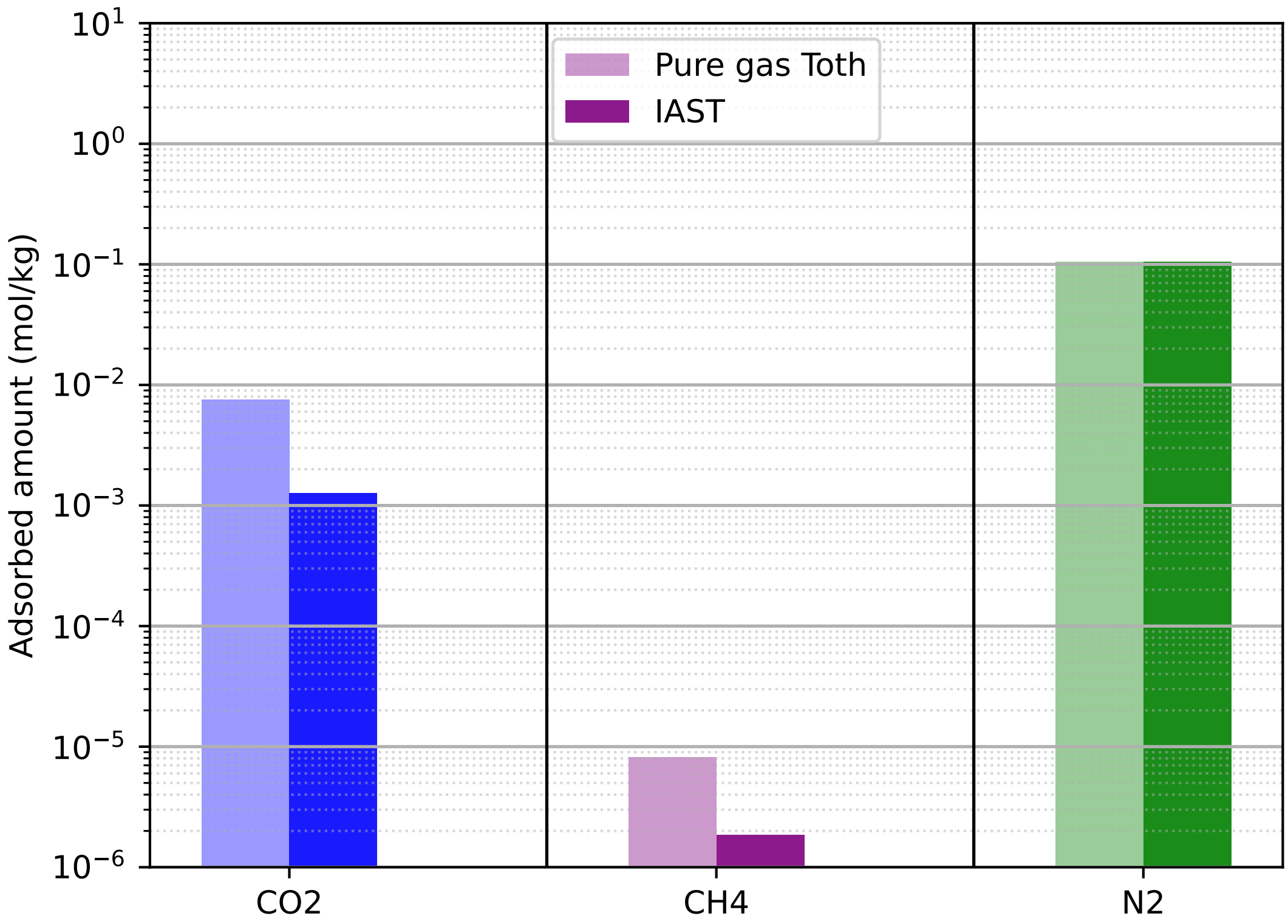

Figure S7: Comparison of pure gas and IAST gas adsorption for air, Cu-MOF(7), 298 K.

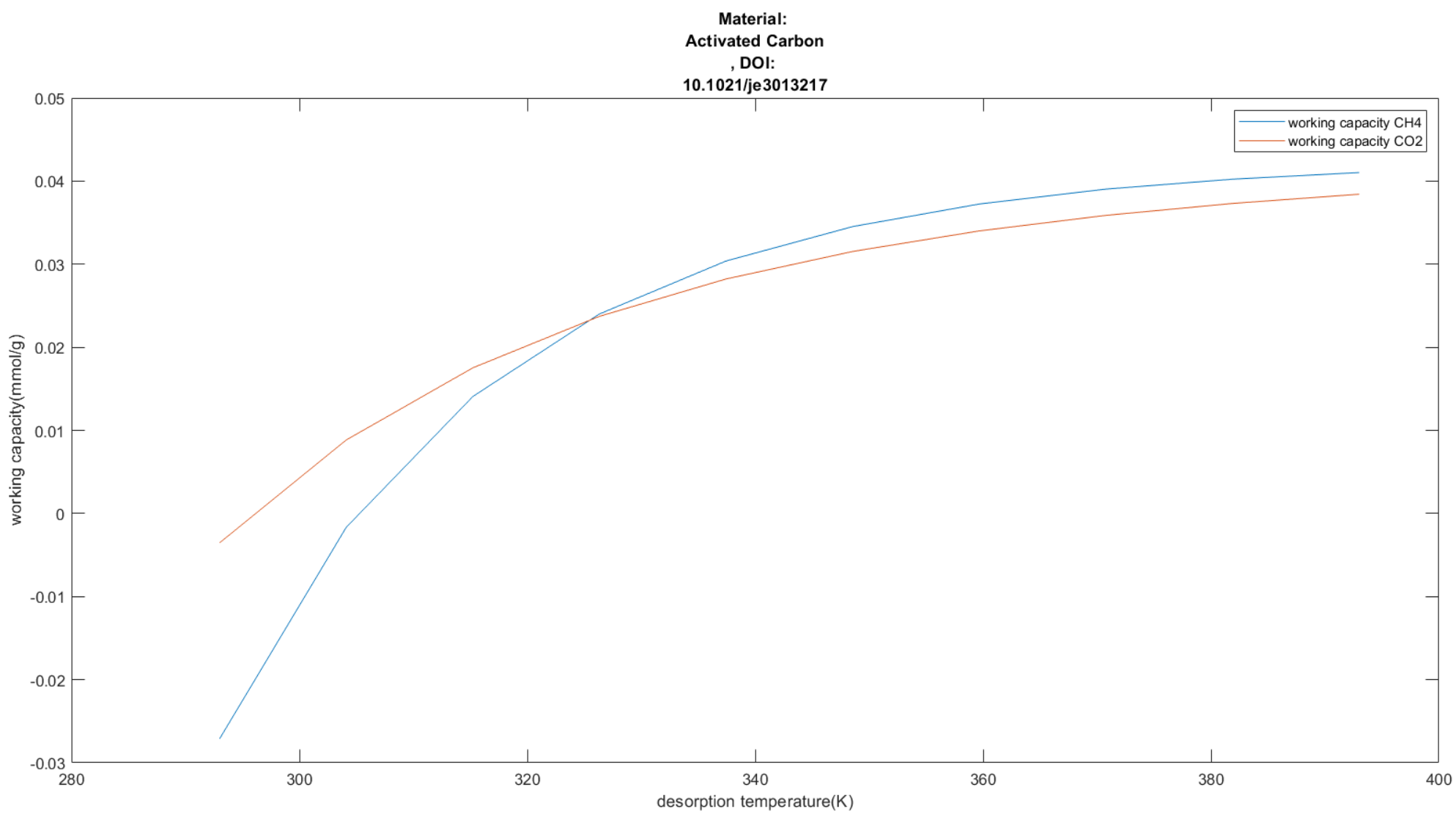

Figure S8: Working capacity between adsorption and desorption for Activated Carbon.

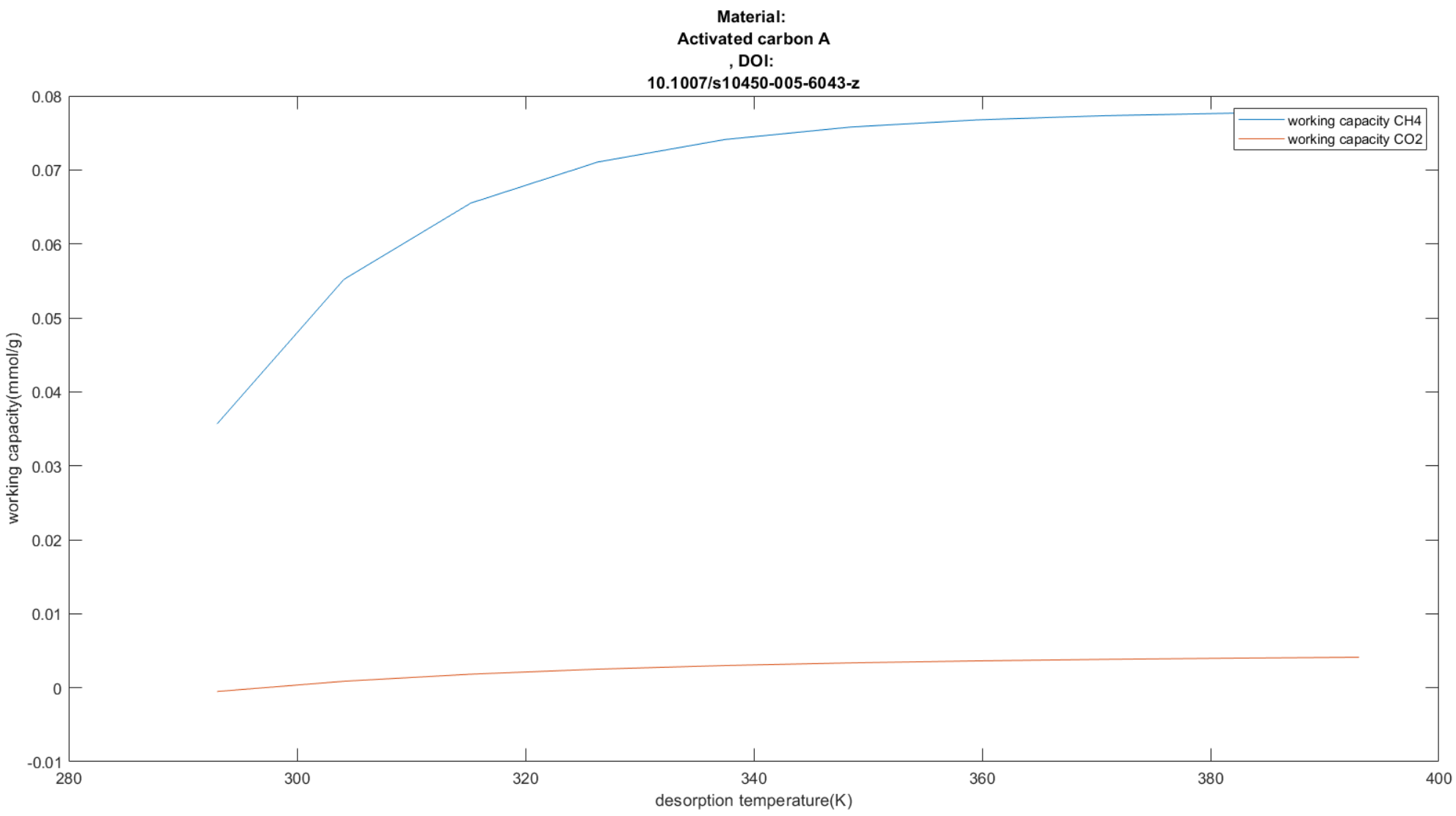

Figure S9: Working capacity between adsorption and desorption for Activated Carbon.

Nomenclature

q, q^*	adsorbed amount, mol/kg^3
i	component
y	mole fraction
p	pressure, bar
T	temperature, K
T_0	reference temperature, K
ΔH	isosteric heat of adsorption, J/mol
R	universal gas constant, J/mol/K
N	Number of mole, mol
k	sub-step
Q	specific heat, kJ/kg
s	solid
m_s	sorbent mass, kg
$c_{p,s}$	specific heat capacity at constant pressure, kJ/kg/K
t_f	final time
t_0	start time
$\dot{N}$	molar flow
amb	ambient
V	column volume, m^3
ϵ	void fraction
t	time,s
u	velocity, m/s
ρ_b	bed density, kg/m^3
n_i	adsorbed amount of component i at time t, mol
x	longitudinal distance, m
k_i	lumped mass transfer coefficient of component i, 1/s

n_i^{eq}	equilibrium adsorbed amount of component i, mol
ϵ_t	total void fraction
C_g	heat capacity of the gas, J/(Km^3)
C_s	heat capacity of the solid, J/(Kkg)
C_{ads}	heat capacity of the adsorbed phase, J/(Kkg)
h	heat transfer coefficient, J/(m^{2}K)
T_w	wall temperature, K
$C_{p,w}$	heat capacity of the wall, J/(Km^3)
d_0	outer column diameter, m
d_i	inner column diameter, m
μ	dynamic viscosity, Pa.s
d_p	particle diameter, m
